# Detecting anomalous referencing patterns in PubMed papers suggestive of author-centric reference list manipulation

**DOI:** 10.1007/s11192-022-04503-6

**Published:** 2022-09-08

**Authors:** Jonathan D. Wren, Constantin Georgescu

**Affiliations:** 1 Genes and Human Disease Research Program, Oklahoma Medical Research Foundation, 825 N.E. 13th Street, Oklahoma City, OK 73104-5005, USA; 2 Biochemistry and Molecular Biology Department, University of Oklahoma Health Sciences Center, Oklahoma City, OK, USA; 3 Stephenson Cancer Center, University of Oklahoma Health Sciences Center, Oklahoma City, OK, USA; 4 Department of Geriatric Medicine, University of Oklahoma Health Sciences Center, Oklahoma City, OK, USA

**Keywords:** Citation behavior, Citation analysis, Scientific ethics

## Abstract

Although citations are used as a quantifiable, objective metric of academic influence, references could be added to a paper solely to inflate the perceived influence of a body of research. This reference list manipulation (RLM) could take place during the peer-review process, or prior to it. Surveys have estimated how many people may have been affected by coercive RLM at one time or another, but it is not known how many authors engage in RLM, nor to what degree. By examining a subset of active, highly published authors (n = 20,803) in PubMed, we find the frequency of non-self-citations (NSC) to one author coming from a single paper approximates Zipf’s law. Author-centric deviations from it are approximately normally distributed, permitting deviations to be quantified statistically. Framed as an anomaly detection problem, statistical confidence increases when an author is an outlier by multiple metrics. Anomalies are not proof of RLM, but authors engaged in RLM will almost unavoidably create anomalies. We find the NSC Gini Index correlates highly with anomalous patterns across multiple “red flags”, each suggestive of RLM. Between 81 (0.4%, FDR < 0.05) and 231 (1.1%, FDR < 0.10) authors are outliers on the curve, suggestive of chronic, repeated RLM. Approximately 16% of all authors may have engaged in RLM to some degree. Authors who use 18% or more of their references for self-citation are significantly more likely to have NSC Gini distortions, suggesting a potential willingness to coerce others to cite them.

## Introduction

Quantitative metrics that reflect the potential impact of a researcher’s work or influence of a journal’s papers are highly preferred over subjective metrics, and citations are typically the most influential. For authors, being well-cited can potentially correlate with tangible rewards such as promotions, tenure, and awards, as well as intangible things such as professional and/or societal respect. For journals, citations correlate with quality of future submissions, prestige of being on the editorial board, and potential revenue. Insofar as citations are linked to rewards, individual entities (e.g., researchers, journals) become incentivized to increase the number of citations to their work. Implicit in this is the assumption that citations, particularly citations that come from others, reflect the impact their work has had on their peers. Gatekeepers in the peer-review system, however, have the opportunity and ability to influence references included in a paper. Similarly, authors may feel motivated to include some references not intended to support key points, but to pay homage to some valued entity (e.g., former advisor, departmental chair, colleague, etc.). Even outside the peer-review system, there may be ways to manipulate citations, such as by creating documents with citations to your target to be indexed by Google Scholar (Lopez-Cozar et al., 2012).

Surveys have attempted to estimate different aspects of reference list manipulation, such as how many authors have been affected by coercive citation practices (Fong & Wilhite, 2017). A 2008 survey of 283 authors found 22.7% of them reported “*a reviewer had required them to include unnecessary references to his/her publication(s)*” (Resnik et al., 2008). Although this survey was relatively limited in its scale and scope, and the key word “unnecessary” does not necessarily imply that the references were inappropriate or excessive, but it does suggest two things: First, by virtue of the number and nature of the citation requests, the author believes they were able to infer the reviewer’s identity. Second, requests for additional citations that, in the author’s view, are not merited may be fairly common in scientific peer-review. It is not clear, though, what fraction of these requests might be characterized as ego-driven (e.g., demanding recognition of their contribution) versus what fraction might be attempts to influence metrics (e.g., H-index). Similarly, another survey found more than 20% of respondents had experienced coercion from a journal editor (Wilhite & Fong, 2012).

Another study reported that 29% of the references that reviewers requested be added during peer-review were to the reviewer’s own work (Thombs et al., 2015). The study, however, did not ascertain what fraction was perceived as unmerited. However, in reviews recommending acceptance/revision, more than twice as many reviewer self-citation requests were found than in those recommending rejection (reported p < 0.001), whereas the number of requested citations to the work of others did not significantly differ. Interestingly, a related study found that requested self-citation frequency did not differ between blinded and open peer-review (Levis et al., 2015), suggesting author awareness of a reviewer’s identity is not a significant disincentive. Instances of various types of citation hacking have been documented, but are hard to detect in general (Ioannidis, 2015; Wren et al., 2019).

The primary purpose of citations is so the reader can unambiguously link the most important aspects of the new research being presented to prior research. How merited a citation is depends upon how much it contributes to the understanding and reproducibility of specific points made within a paper. If citations are included in a paper solely to increase someone’s perceived influence, this is deceptive at best. If an editor or reviewer were to use their trusted position within the peer-review process to increase the perceived influence of their own work or of a body of work they have a vested interest in, this is a conflict of interest and unethical behavior. When peer-review gatekeepers request *unnecessary* citations to their own work or journal, this has been referred to as coercive self-citation (Thombs et al., 2015; Wilhite & Fong, 2012). For journals, the motivation would likely be to improve their standing (e.g., impact factor), for which the manipulator could obtain either direct or indirect benefits (Huggett, 2013). Authors including unmerited citations (to work other than their own) could be the result of a favor or a *quid pro quo* arrangement between authors which, when recurring, are referred to as “citation rings” (Biagioli, 2016; Chen et al., 2012). Editors influencing unmerited citations may be a result of coercion but, depending upon the manuscript handling process, it’s also possible they could simply insert citations to their own work after a paper is accepted without the knowledge of the authors. Unmerited journal self-citation can be achieved either by encouraging authors to cite more papers from their journal (Wilhite & Fong, 2012) or simply by publishing content with self-citation. When journals unified by a common interest (e.g., same parent company or editor) cite each other, this is called “citation stacking” (Heneberg, 2016). There has been a fair amount of work in detecting anomalous journal citation patterns algorithmically. For a more thorough overview, Ioannidis has written a very helpful commentary discussing the many different types of self-citation, depending on where they originate and how they manifest (Ioannidis, 2015). Each of these types of behavior is known by a different name, but are all related by a common theme: Manipulation of reference list contents to artificially increase the perceived influence of some entity.

The goal of this study is to develop and test a method to detect patterns of potential reference list manipulation (RLM) by individual authors. This is with the caveat that we are using a statistical approach, and is not based upon a gold-standard of empirically determined instances of RLM. We thus propose the term “citation hacking” may be a more descriptive term for this. Hacking, as a phenomenon, involves unauthorized, non-transparent access to a system that is otherwise presumed secure and/or trustworthy, such as a publication that has passed peer-review and is presumed to be a product of the authors altered only by good-faith feedback during peer-review, to perform some action that benefits the hacker. Although in theory, citation hacking might encompass actions such as vandalism or inserting web links to download viruses, in practice we expect the goal of virtually all instances would be to increase the perceived influence of a body of research. Although the most likely beneficiary would be the citation hacker, either directly (to their own research) or indirectly (to journals they are founders of or editors for), it’s conceivable that the intended beneficiary could also be a group of people united by some common factor (e.g., university, department, co-investigators on a grant, etc.), or even a favored theory that is in dispute. Thus, the term “citation hacking” encompasses the use of non-transparent means to achieve an end goal (to increase perceived influence) within a specific system (publication & peer-review), but is agnostic to the means used and entity responsible. By this definition, self-citations (SC), because they are transparent to readers, are not citation hacking.

There are three important issues that motivate this study. First, estimate the prevalence of citation hacking in PubMed. Although surveys have approximated how many authors have been victims of coercive citation, it is not known how many researchers may have engaged in citation hacking and to what degree (e.g., many to some extent, a few to a large extent, or both). Related to this point, it is also of interest to identify potential risk factors that might predict future behavior. Second, provide and evaluate a relatively simple and interpretable metric that could be used to identify potential citation hackers. As we noted in a previous publication (Wren et al., 2019), even after citation hackers are discovered, because of privacy concerns and a highly decentralized publishing system, there is no effective mechanism to share information. Even though there are checks-and-balances built into the peer-review system (e.g., editors screening reviewer reports), they are often only witnesses to a single incident and reluctant to raise concerns on that basis alone (Martin, 2013). Furthermore, there is little incentive to publicly disclose citation hacking events once uncovered. In fact, there are potential disincentives such as reputational embarrassment and potential litigation risks if naming offenders. Given the large, decentralized ecosystem of journals, citation hackers can potentially continue unabated simply because the number of people aware of their behavior within this ecosystem will be a minute fraction of the whole. Third, under the presumption that most reviewers requesting self-citation, even when excessive, would rationalize their request in well-intentioned but non-falsifiable terms (e.g., “it will improve the paper”), the only objective option at this point is to put both the individual request and reviewer’s past behavior within the context of their peers. If an individual incident is well beyond the norm and the reviewer has exceeded the norm on multiple occasions, this is evidence of a pattern, but drawing a conclusion requires a reference for what defines ‘normal’ behavior.

## Methods

### Obtaining citation data

PubMed 2020 records were downloaded in XML format on May 25, 2020 from NCBI (ftp://ftp.ncbi.nlm.nih.gov/pubmed/). For each paper, we extracted its PubMed ID (PMID), all author names, name of the journal it was published in, the journal’s ISSN, and the PMIDs of each paper within the reference list, when given. Only references that contain a PMID are included in the citation network (i.e., a paper may have more total references than the ones extracted).

**Fig. 1:**
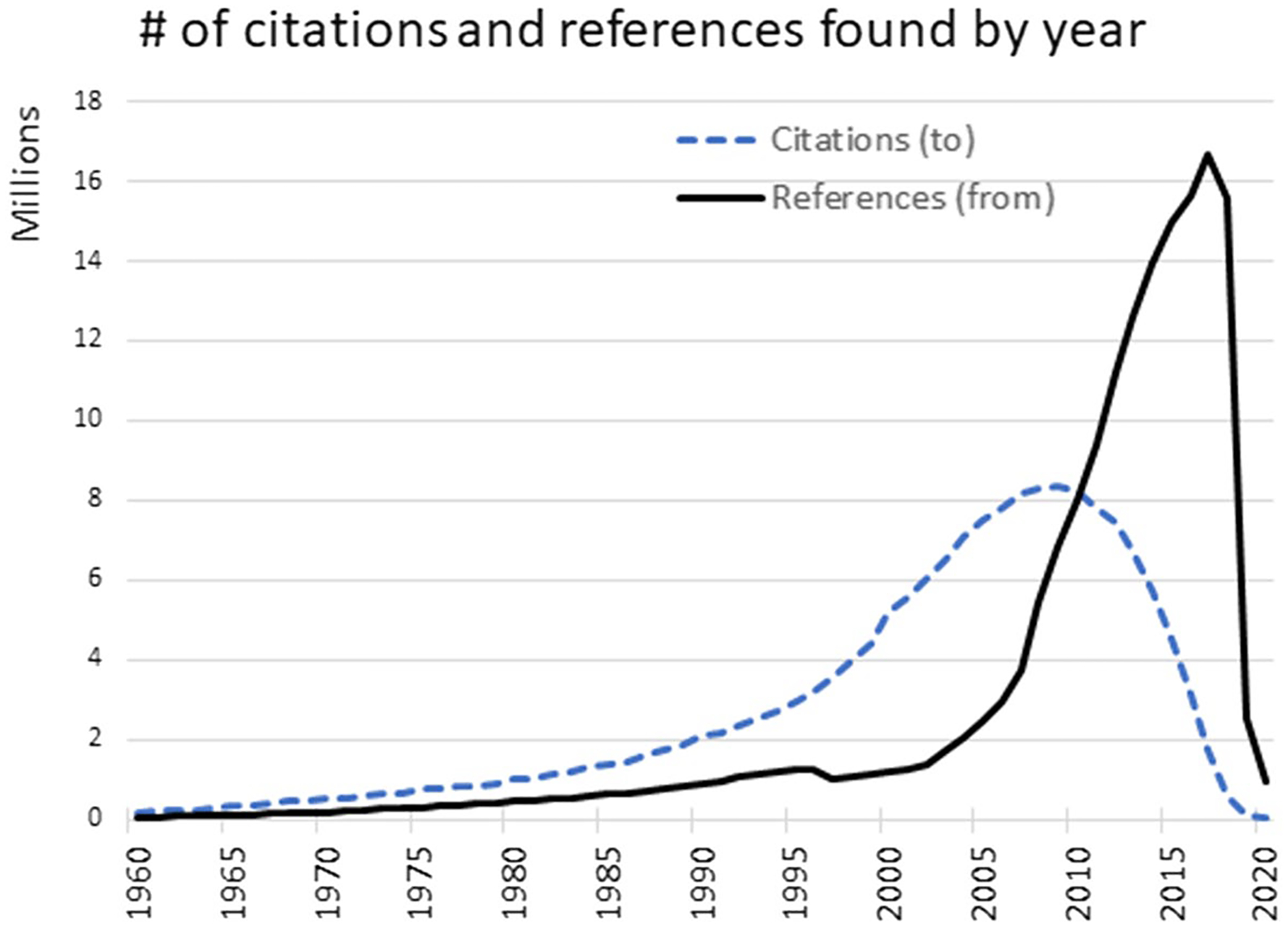
The total number of references found in PubMed papers by year (black line). The dashed blue line shows the years in which the cited publications were published. There is an apparent lag time to entry of citation data, so citations to the most recent papers are sparse, which appears as a sharp drop-off

Figure 1 shows the distribution, by year, of citations to papers and references from papers. Since references are extracted from papers deposited in PubMed Central (PMC), they are heavily biased towards more recent papers, although citations to papers extends much further back. Within this dataset there were 31,029,833 unique PMIDs, 6,003,225 (18%) of which contained at least 1 reference, and 16,338,882 (53%) of which received at least one citation. A total of 172,528,049 PMID-PMID citation links were identified.

References in the XML records tend to be given in order of their appearance within the full text, even if the journal’s publication reference format is alphabetical by author name. This enables analysis of over-represented author names within blocks of contiguous references, but we note that, upon comparing several extracted lists with the PDF of the publication, there were some discrepancies in the ordering, suggesting the mapping is approximate, not exact. Thus, it is problematic to accurately quantify, using this data, the largest contiguous block of citations to one author, but less problematic to estimate how many smaller contiguous blocks exist.

### Subsetting authors for analysis

We restricted our author list to include only authors who have published recently (most recent publication within the citation network no older than 2017) and to those who authored or co-authored at least 100 papers within the citation network (i.e., 100 papers with at least one citation to or from the paper). We denoted authors in the first and last positions as “anchor” authors, because they are considered to have had a disproportionate influence on the content of the paper (Wren et al., 2007). When we refer to “authorship”, this includes co-authorship, and is defined simply as the presence of an author’s name on the authorship byline. Accented letters were converted (e.g., “Peña” = > “Pena”) to reduce potential inconsistency in transcription (e.g., by a co-author when writing, a journal when formatting, or PubMed when indexing).

Because our analysis is author-centric, in the absence of a widespread unique author identifier within the metadata (e.g., ORCID), it was necessary for us to attempt to reduce the level of author name ambiguity. We did this by restricting analysis to authors with names that included at least one middle initial or, if not, then had either two first or last names (as judged by the presence of either a hyphen or space within the first/last name). Also, we required their middle plus first name to be at least 4 characters in length. A total of 20,803 authors fit all these criteria. One downside to this limitation is that it will underestimate the total number of citations to authors in proportion to the number of inconsistencies in their full name (e.g., “Smith, JA”, “Smith, John Abrams”, and “Smith, John A” would be counted as different people even if they refer to the same person), or if the author underwent a name change at some point (e.g., due to marriage). Also, because authors tend to cite more of their own papers than papers from another author, median SC frequency from individual papers is higher than median NSC frequency. Thus, there is a risk that highly self-citing authors that are also highly inconsistent in the spelling or structure of their name will be detected as having highly distorted NSC (i.e., many will actually be misclassified SC). Ambiguity of author names, by these criteria, appears to be more of a problem prior to 2002 than after, perhaps because of a shift from recording author first/last names as initials to their full-form. It is difficult to estimate what fraction of the whole this subset represents because, although there were over 12 million unique author name strings identified, some authors will share a name. Furthermore, ~ 46% only occur once, some of which will be attributable to name variations (including spelling errors), and others to people who have only published once.

### Normalization of the “red flag” metrics

Many of the proposed red flag metrics scale with an author’s total number of non-self citations (NSC). To more effectively remove the influence of total NSC, we subtracted out the effect of total NSC by modeling the relationship between the two variables using robust linear regression, in log–log space. Log transformations were determined using the box-cox power transform to be optimal for meeting standard regression model assumption requirements, including linearity, normality and homoscedasticity. Exponentials of the regression residuals return the observed vs expected ratios, and are shown in graphs as normalized values. In raw form, in log–log space, the regression residuals are approximately normally distributed, providing suitable inputs for Factor Analysis.

## Results

### The number of non-self-citations (NSC) to one author from one paper approximates a Zipfian distribution

**Fig. 2:**
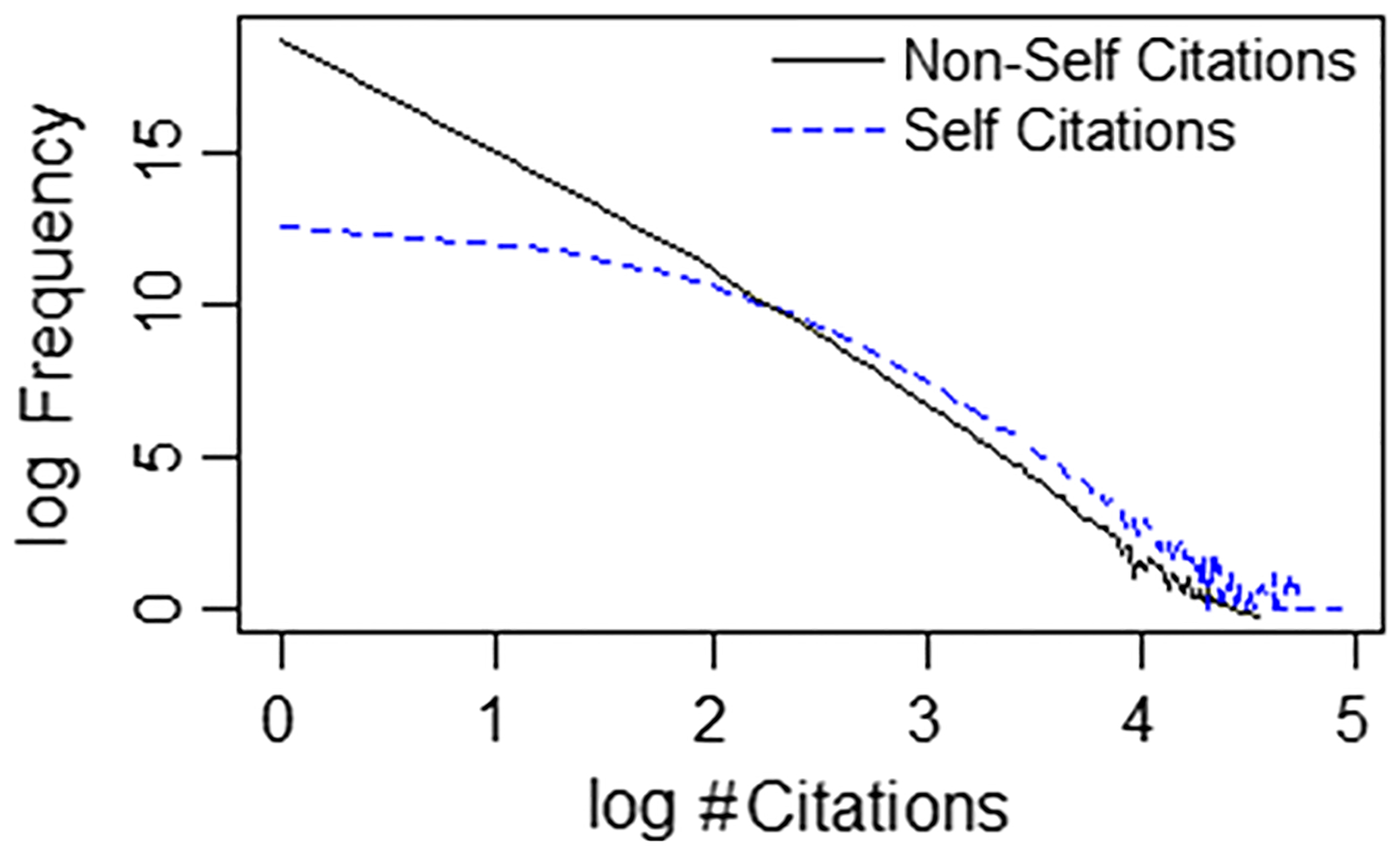
Cumulative # of times (y-axis) a single author within our subset was cited within a single PubMed paper exactly *n* times (x-axis), plotted as the natural logarithm of the number. NSC = non-self citations, SC = self-citations

Citation hacking, by definition, takes place on the level of the individual paper. The frequency of citations, within one published paper, to any of a single author’s entire body of published papers, is approximately power-law distributed or Zipfian. For our data, we find linear projection (Ordinary Least Squares) of NSC frequency on NSC number, on a log–log scale explains more than 90% of variability (R^2^ > 0.9) for more than 95% of the authors. Figure 2 shows this linear log–log relationship, characteristic of a Zipfian distribution, is roughly valid overall, for our author subset. Self-citations (SC) did not follow a Zipfian distribution, but this was expected since they are governed by different mechanisms (i.e., an author’s preference as opposed to external awareness/interest).

Zipfian distributions are known to arise in a variety of natural systems and are thought to be governed by laws of preferential attachment (Ghosh et al., 2014; Schwab et al., 2014). An important implication of Zipf’s law to this study is that, because the trend is linear in log-space, future values in the series can be approximated using the initial or early values in the series. Thus, the frequency by which any author has received *n* citations from a paper should be proportional to the frequency by which they receive *n* + 1, *n* + 2, etc. This frames the problem overall by defining statistical expectations regarding what is “normal” and enabling null hypothesis testing for observed frequency distributions. It does not mean that authors with skewed distributions have necessarily engaged in RLM, but it does mean that all authors who do engage in RLM will have skewed distributions in proportion to their activity. The more references requested per attempt and the more frequent the requests, the more severe the skew.

### Identifying “red flags” suggestive of citation hacking

Detecting citation hacking is complicated by several factors: First, the fraction of all authors regularly attempting to influence citation of their work to a large degree during peer-review is presumed to be rare. Second, there is no gold-standard or ground truth to evaluate how well a metric reflects citation hacking activity. Third, citation hackers may have different strategies and/or opportunities to influence reference lists. Finally, accusations of citation hacking would be a sensitive matter and, consequently, more than one line of evidence is highly desirable. These are common circumstances in the field of anomaly (or outlier) detection. One method of gaining confidence in declaring a data point an anomaly is for it to be anomalous by several different measures attributable to a common cause (Agrawal & Agrawal, 2015). For example, readings across multiple sensors within a device are often used to diagnose a potential root cause (e.g., overheating, electrical surge, etc.). So, we examined several citation pattern “red flags” that are suggestive, but not independently conclusive, of citation hacking (Table 1).

Each flag is motivated by economic considerations: If someone wants to increase their perceived influence via increased citation of their work, but their supply of opportunities is limited, then their incentive is to maximize the number of references added per opportunity available. This is mitigated by other factors such as author compliance and editorial intervention, but also by some expectation the hackers have regarding potential costs associated with their behavior being called into question.

### The Gini Index as a proposed metric for quantifying skew in a frequency distribution

**Fig. 3:**
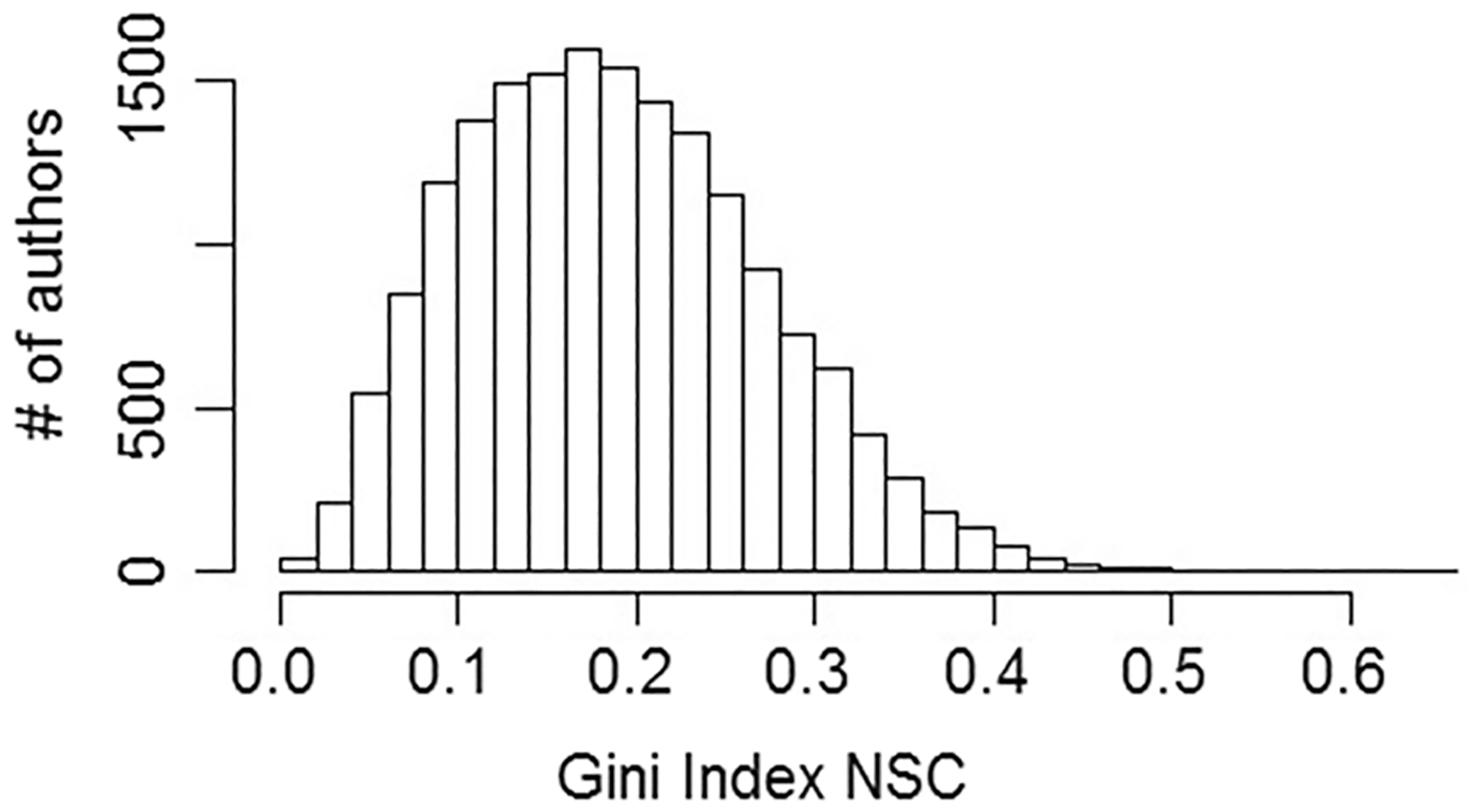
Distribution of Gini Index values (mean = 0.185 ± 0.084), quantifying how skewed the single-paper NSC frequency distribution is for each author. The higher the Gini, the more often an author had unusually large numbers of NSC to their work coming from single papers, relative to their overall distribution

The Gini Index is a well-known statistical measure of dispersion and inequality (Dodge, 2008). It is especially popular in economics to quantify inequalities of income distribution. Formally, the Gini coefficient is proportional to the area between the cumulative changes of a variable of interest across a population normalized to a percent value (1%−100%), and a straight line which represents a uniform distribution. Gini values range from 0 (perfect uniformity) to 1 (one observation contains all the values). The Gini coefficient is half of the relative mean absolute difference of the variable under study (Sen et al., 1997). For an author cited at least once within *n* papers written by other authors, where x_i_ is the number of non-self citations (NSC) to that author given within the ith paper (i = 1,…,n) and x_j_ the NSC from the j_th_ paper (j = 1,…,n), the Gini coefficient can be computed as:

$$G=\overset{n}{\underset{i=1}{\sum }}\overset{n}{\underset{j=1}{\sum }}\left|x_{i}-x_{j}\right|/2n^{2}\bar{x}$$

where $\bar{x}$ is the mean NSC per paper. A friendly implementation of the formula is provided by the function “Gini” within the ineq R package. Thus, the Gini will be a measure of how skewed the NSC distribution is for each author across all papers referencing any of that author’s entire body of work. If all papers received the same number of citations the Gini would be zero (perfect uniformity), and if all the citations came from one paper it would be one (maximally non-uniform). One advantage of the Gini is that, as a scale-independent relative measure, it does not require normalization. The distribution of Gini index values for all authors is shown in Fig. 3.

To examine how sensitive the Gini is to outliers, we removed the paper with the most NSC for each author, recalculated their Gini and compared the rankings using Spearman’s correlation. We found a high concordance (R = 0.996) and that within the top 1% highest Gini’s (n = 208), the most an individual author dropped was 32 positions, suggesting their membership at the extreme end was not sensitive to single outlier removal.

We also wanted to examine whether or not the Gini might be influenced by “mega-reviews”. Mega-reviews are papers with an unusually high number of references that attempt to summarize work within a large area of research. Thus, they might be prone to citing individuals in the field more frequently in a single paper. Since there is no standard definition for how many references make a paper a mega-review, we recalculated Ginis after excluding NSC from all papers with > 150 references, which encompasses only 0.8% of all papers but 6.3% of all references. Within the top 1%, one author’s Gini fell by a striking 788 positions, but the average drop for the remaining authors was only 1 rank. Further examination of this author shows 128/991 (13%) of the papers with > = 1 NSC to them also had > 150 references, suggesting 150 may be an insufficiently small cutoff for some fields, and showing that this drastic change in Gini was not attributable to a small number of mega-reviews.

**Fig. 4:**
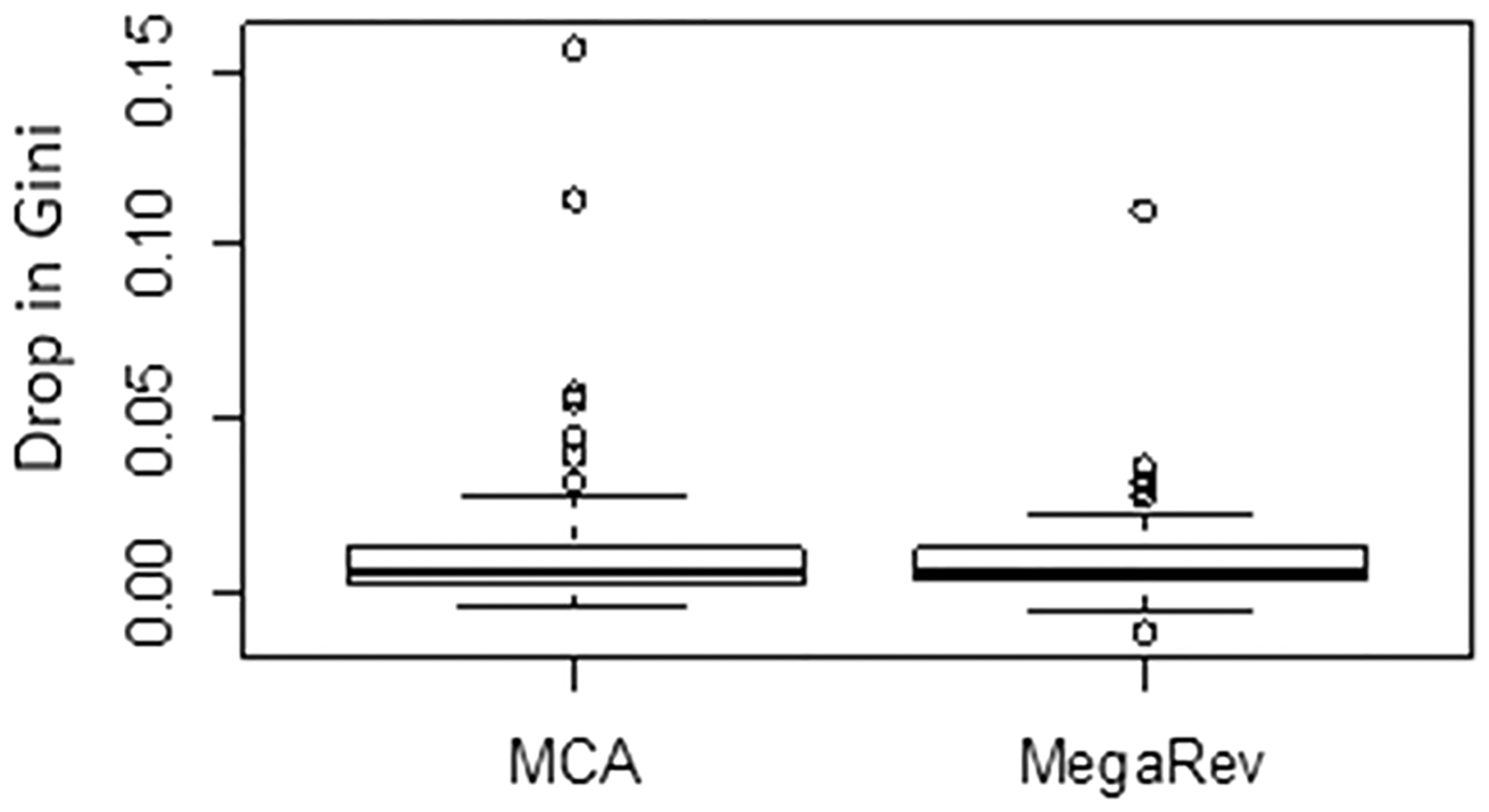
Drop in Gini for authors with the highest 1% NSC Gini Index scores (n = 208, avg NSCGI = 0.434 ± 0.035) when subtracting all papers published by their Most Citing Author (MCA) or all mega-reviews (MegaRev, defined as papers with > 150 references)

Finally, we estimated how much their Most Citing Author (MCA) affected the Gini of each author in the list, as defined by an H-index-like measure of at least *n* NSC observed in at least *n* papers for each citing author. Within the top 1%, the ranking for two authors dropped substantially (Fig. 4), but most did not change appreciably. Note that Gini contributions from single outliers and mega-reviews are viewed as potential confounds, but are not necessarily innocuous. For example, an MCA simply be an admirer or a *quid-pro-quo* might exist, and a mega-review might simply reflect an author’s specialization within the topic of review or the emphasis on coverage may have created the impression for a reviewer/editor that more of their work qualifies. Based on string similarity of each author’s name versus their MCA, we estimate that about 1.4% of the authors have an MCA that is actually a variation on the spelling of their own name. This suggests that mistaking SC for NSC is happening at a fairly low rate.

### Red Flag #1: seeing large and/or frequent blocks of consecutive NSC to an author within a paper

**Fig. 5:**
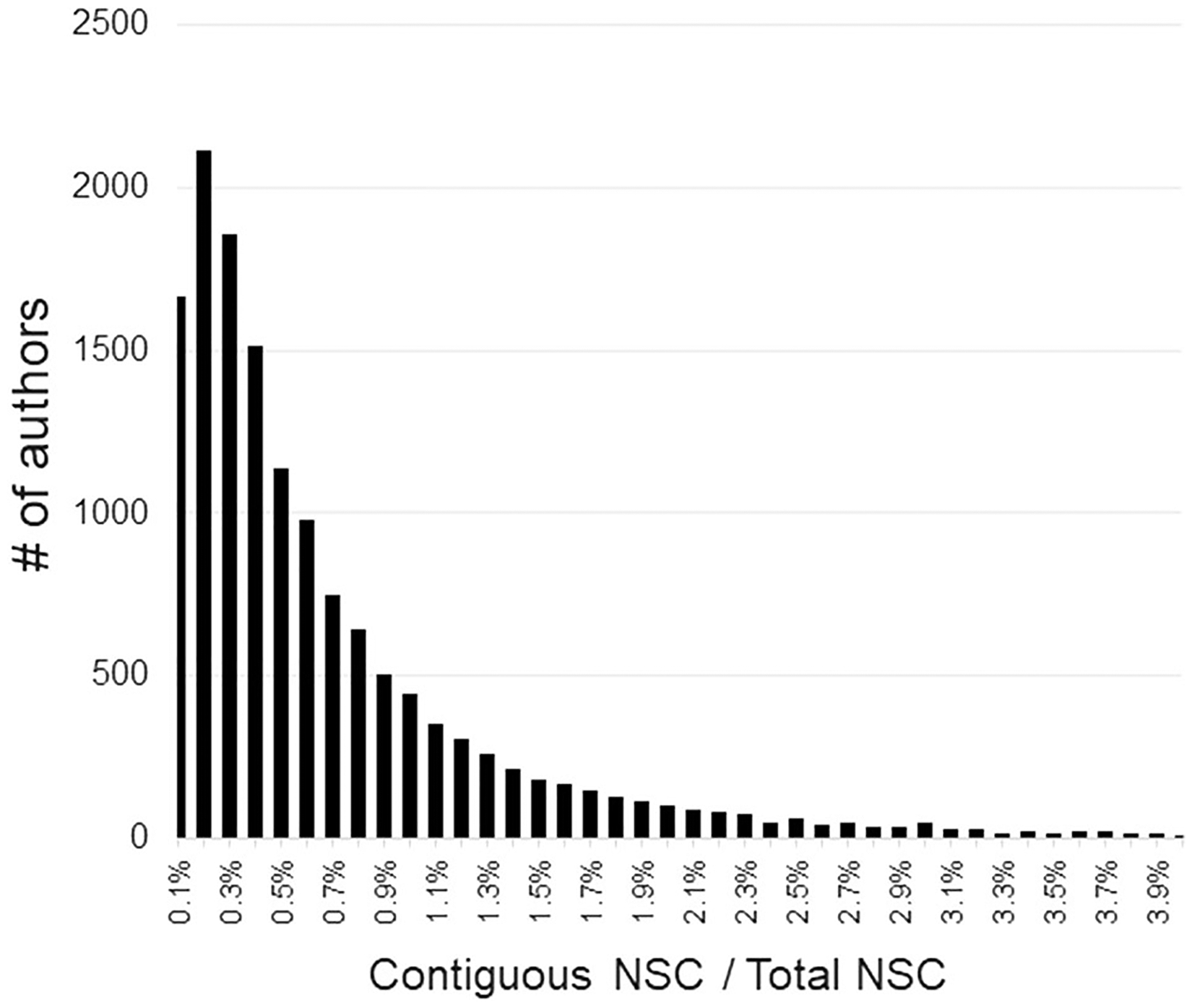
Histogram showing what fraction of the total NSC to an author came from blocks of at least 3 consecutive references to their work found within a single paper. The *x*-axis is truncated at 4% (the maximum value observed was 64.4%). For the large majority of authors, blocks of consecutive citations to their work are infrequent events

References are expected to support the topic at hand and, although there may be valid reasons for an author-centric series of consecutive references, it is not the norm. Unusually large and/or frequent blocks of contiguous NSC are highly suggestive authors may be accommodating a request from either a reviewer or editor. Not only did we observe this for the coercive reviewer documented in our case report (Wren et al., 2019), but it makes sense that when authors are adding citations solely to satisfy a reviewer’s concerns, they would generally do it in one or possibly a few blocks of consecutive citations. The alternative would be to try to weave them throughout the text and, particularly for unmerited citations, would be quite difficult to do in a way that appeared natural or logical to the reader. Thus, we expect that a common “fingerprint” left by citation hackers would be large blocks of contiguous citations to them within a paper. This metric lends itself to validation by examining the surrounding context of citation blocks. The more generic the statement (e.g., “other work has been done in this area”) and the larger the block, the less likely the citations were motivated by the topic or necessary to the paper. Considering authors with at least 200 NSC (n = 20,712), the ratio of total consecutive NSC (3 minimum) to total NSC shows that such citation blocks are relatively uncommon events in general, with 30% of authors having none at all (Fig. 5).

### Red Flag #2: multiple papers with an unusually large number of NSC to an author relative to their total NSC

**Fig. 6:**
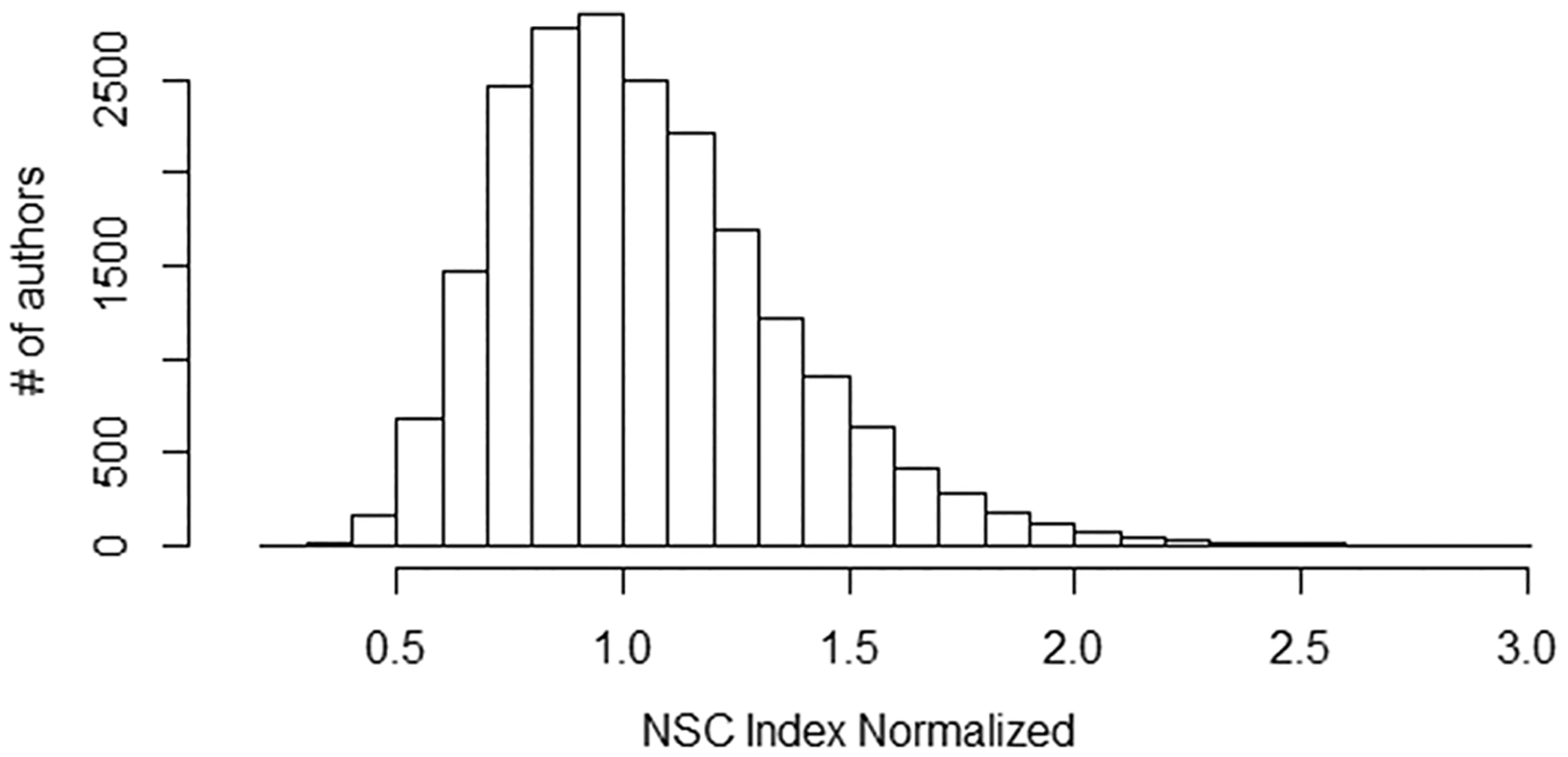
A normalized H-index like measure for NSC coming from individual papers to any one of an author’s papers shows a central tendency, but with a skew towards higher values

Supplementary Table S1 shows the probability of observing n citations to one author’s work in a paper, both NSC and SC. For example, although it is rare to see more than 5 references to someone who is not an author of the paper (~ 1%), it is fairly common to see more than 5 citations to one of the paper’s authors (23%). Thus, conceptually similar to the H-index, we can calculate an NSC Index (NSCI) using the number of times, *n*, that at least *n* NSC came from one paper to a specific author. Because the H-index correlates with the square root of an author’s total citations (Yong, 2014), the NSCI is normalized (see methods). Figure 6 shows the distribution of NSCI values for all authors.

### Red Flag #3: papers that contain an extremely large number of NSC to an author’s body of work

The NSCI, similar to the H-index, seeks to discount the extreme end of the citation curve in favor of a metric that more stably reflects the entire distribution. However, extreme events are not only informative, but reflect how egregious the hacker can be and also represent instances whereby the peer-review system has clearly broken down. For example, if someone coerces the insertion of 49 references to their work, the NSCI could detect this if evenly spread out (7 papers with 7 NSC each), but not if they are all in one paper. Similarly, it seems less concerning to discover an editor did not question or notice a reviewer requesting 7 self-citations in 7 separate reviews spread out over time versus an editor who did not question or notice a reviewer who requested 49 self-citations in one review. Whereas the NSCI prioritizes consistency over egregiousness, the observed to expected ratio of extreme events prioritizes egregiousness over consistency. Note: There may be valid reasons for a paper to contain an extreme number of NSC to one author (e.g., honoring a retired or deceased author). However, we expect this to be relatively infrequent for most authors, but much more common among citation hackers.

**Fig. 7:**
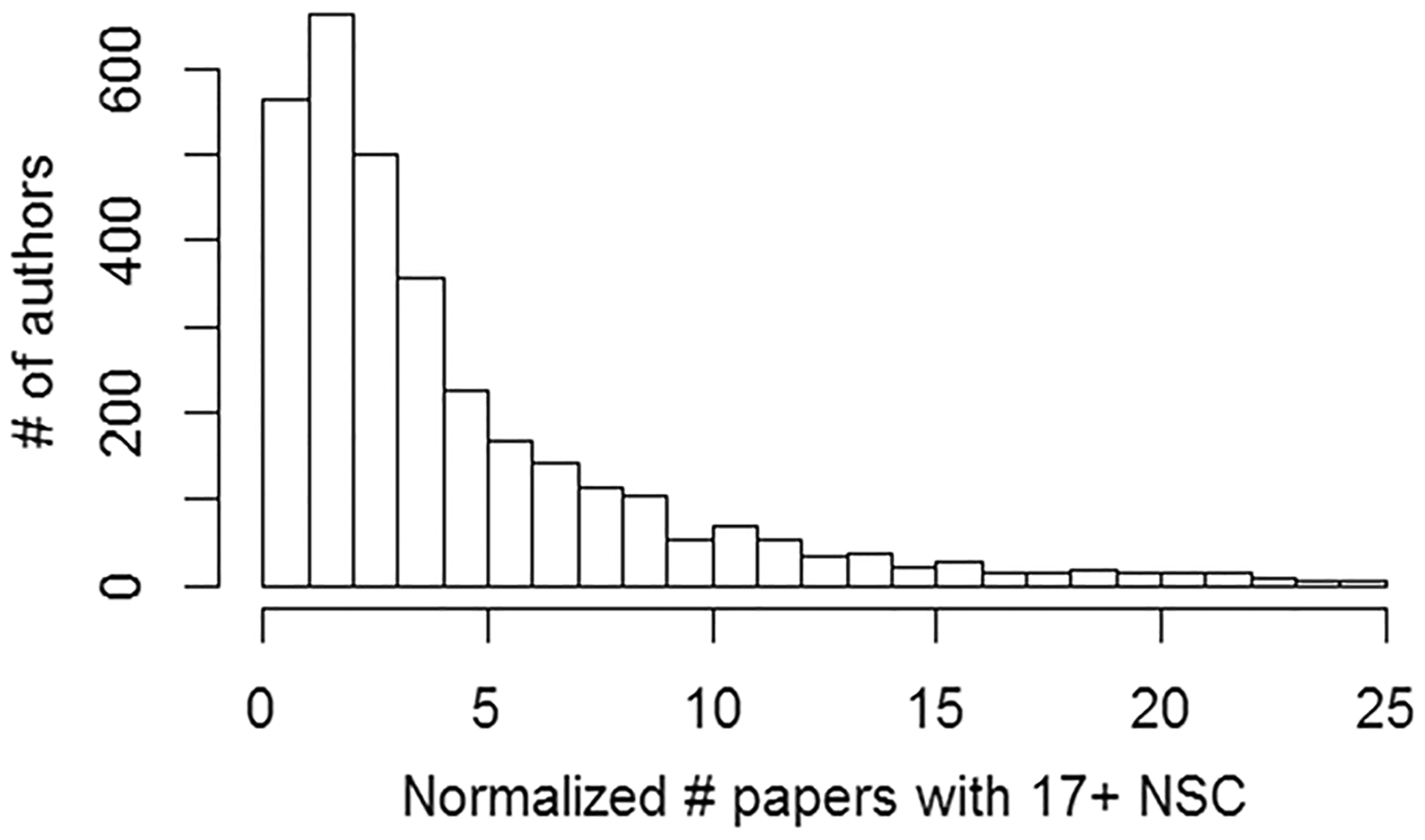
For each author, the number of times an extreme citation event within a single paper is observed (“extreme” defined as 17 or more NSC) is compared to the number one would naively expect, based on how frequent such an event is among all authors in the subset of highly published authors

As Supplementary Table S1 shows, the odds that 17 or more NSC will come from one paper to one author is approximately 0.025%, or once per 3,942 papers that contain at least one NSC to an author. 17 was chosen simply because it is a fairly high threshold such that observing 17 + NSC from a single paper to a single author would be an uncommon event for the average author. Indeed, 82% of authors in our subset have never even received 17 + NSC. A total of 12,110 instances of 17 + NSC to one author within one paper were observed within our subset These 12,110 instances contained a total of 261,067 citations (avg = 22). For authors with at least one 17 + NSC, an expected number of such events is computed by modeling the increase with total NSC using Poisson regression (Fig. 7).

### Red Flag #4: an unusually large number of NSC to one author coming from papers published within one journal

**Fig. 8:**
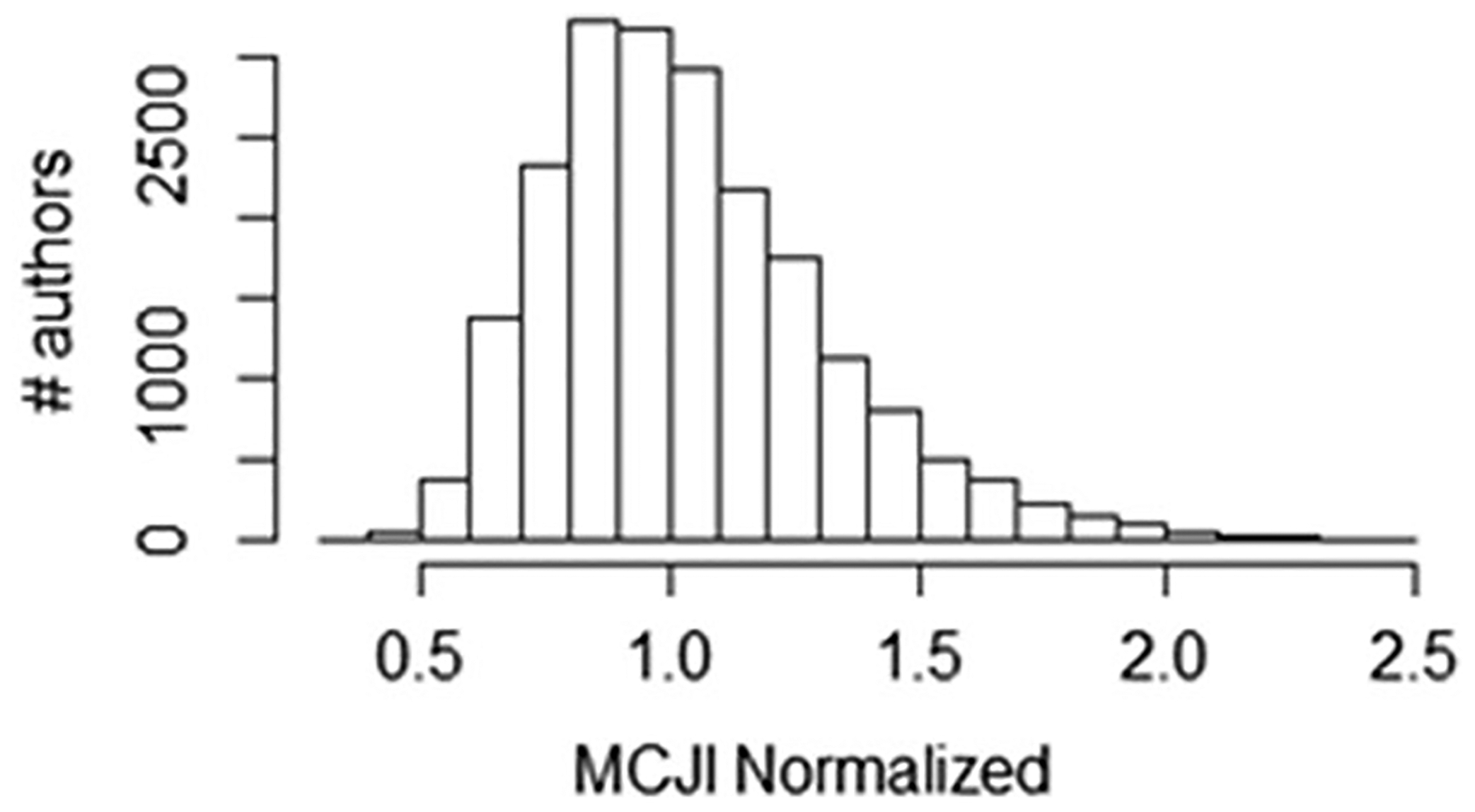
A normalized H-index for citations to an author coming from individual papers published within a single journal is calculated and shows a central tendency. MCJI = Most Citing Journal Index

A high number of NSC to one author from papers published in a specific journal are suggestive of a researcher who may have requested citations to their work in their capacity as handling editor or, possibly, a reviewer frequently used by an editor. First, for each journal publishing a paper with at least one NSC to an author, an H-index like measure, MCJI, is calculated reflecting the largest number of papers, *n*, whereby at least *n* NSC were observed. The journal with the highest *n* is denoted here as the Most Citing Journal (MCJ) for that author. Figure 8 shows the distribution of MCJI values normalized to author’s total number of NSCs from MCJ journal.

Note: Although a high MCJI may be informative, a low MCJI could reflect lack of editorial appointments or reluctance to use that venue for citation hacking. For example, unlike reviewers, editors lack anonymity and their requests would be seen by both authors and reviewers. In fact, a recently published case of an editor using his position to coerce reviewers into citing his papers found he created email pseudonyms so that requests to cite his papers appeared to be coming from a reviewer (Chaplain et al., 2020).

### Red Flag #5: self-citation at the cost of excluding field coverage

**Fig. 9:**
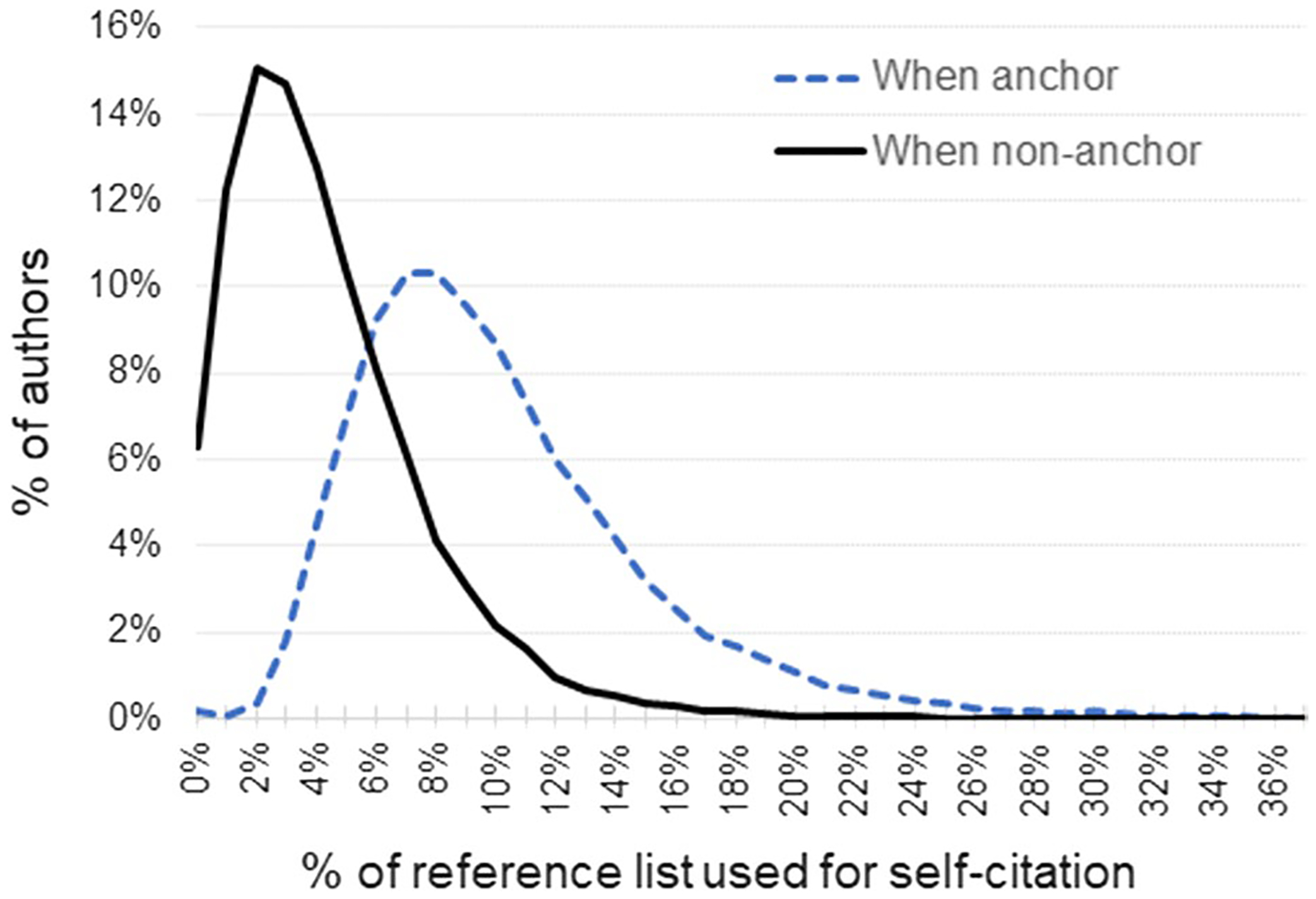
Average percent of the reference list within a single paper used for self-citation within our author subset (x-axis truncated at 36%, maximum value = 66%). Self-citations are more common when an author is either the first or last author on the paper’s byline (i.e., “anchor’s” one side of the byline)

Excessive self-citations, as measured by the fraction of space reserved in an author’s reference list for self-citation, are suggestive that an author is not merely attempting to draw attention to their prior work but, more specifically, using the publication opportunity to maximize the total number of citations to their work. SC are generally transparent and can be subtracted from metrics such as the H-index, when desired. But given that SC may or may not be subtracted, for someone who wants to increase the perceived influence of their work, there is no reason to restrict their efforts to only papers they handle during peer-review, particularly when there is no consensus on whether or not excessive self-citation is an ethical breach (Ioannidis et al., 2019; Van Noorden & Singh Chawla, 2019). Thus, there is potential reward without much risk. Figure 9 shows the distribution of fractional self-citation per author, for authors with a minimum of 9 “anchor” author (i.e., 1^st^ or last author) papers with at least 100 total references among them, broken down by anchor vs middle.

### Gini captures 95% of the ranking information from other metrics

Examining the correlation structure of each red flag metric (Table 2) shows each one contains information about the others to some degree, but are not so highly correlated that they are redundant. While correlations among the first five NSC-based variables were expected, we found it interesting that authors inclined to use a large portion of their reference list for self-citations (%SC) also tended to have distortions in NSC (i.e., from papers they did not author and should, in theory, have no control over).

Factor analysis, similar to Principal Component Analysis, searches for one or more latent/unobserved variables that best explains joint variation within a group of variables. Here, the first latent factor accounts for 55.4% of the group’s total variation, and the second factor only 11%. Factor scores for each variable suggest how well they reflect the behavior of the group. Notably, Gini has the highest predictive power (bold), explaining 51.3% of the first latent variable. This, plus its relative simplicity and the fact it does not require normalization, makes it a good metric to detect potential citation hacking. Supplementary Figs. 1, 2, 3, 4, 5, 6, 7 show plots of each factor versus the others, normalized and non-normalized.

### Estimating levels of chronic and acute citation hacking

The Gini coefficient can be written in many different forms. For example, Lubrano showed Gini can be written as a scaled mean of absolute differences and that the Gini coefficient could be seen as the covariance between a variable and its rank (Lubrano, 2013). Covariance is itself a mean that converges to a normal distribution. Under the assumption that the distribution of NSC Gini index values is approximately Gaussian, which is supported by prior studies (Ultsch & Lotsch, 2017), we can estimate two things. First, we can assign a statistical confidence by which we can reject the null hypothesis that an author’s Gini index value is part of the normal distribution. Chronic citation hackers who engage in repeated and/or egregious RLM would be expected to fall well outside the norm. Second, under the additional assumption that authors would rarely ask for removal of references to their work from papers they review or handle as editor, then in a world where RLM did not exist, the left and right-hand sides of the curve should be symmetric. Because we do know, at least from a limited number of reports, RLM has happened, then the extent to which the real-world curve is right-shifted relative to the ideal curve provides us with a quantitative estimate of the difference.

**Fig. 10:**
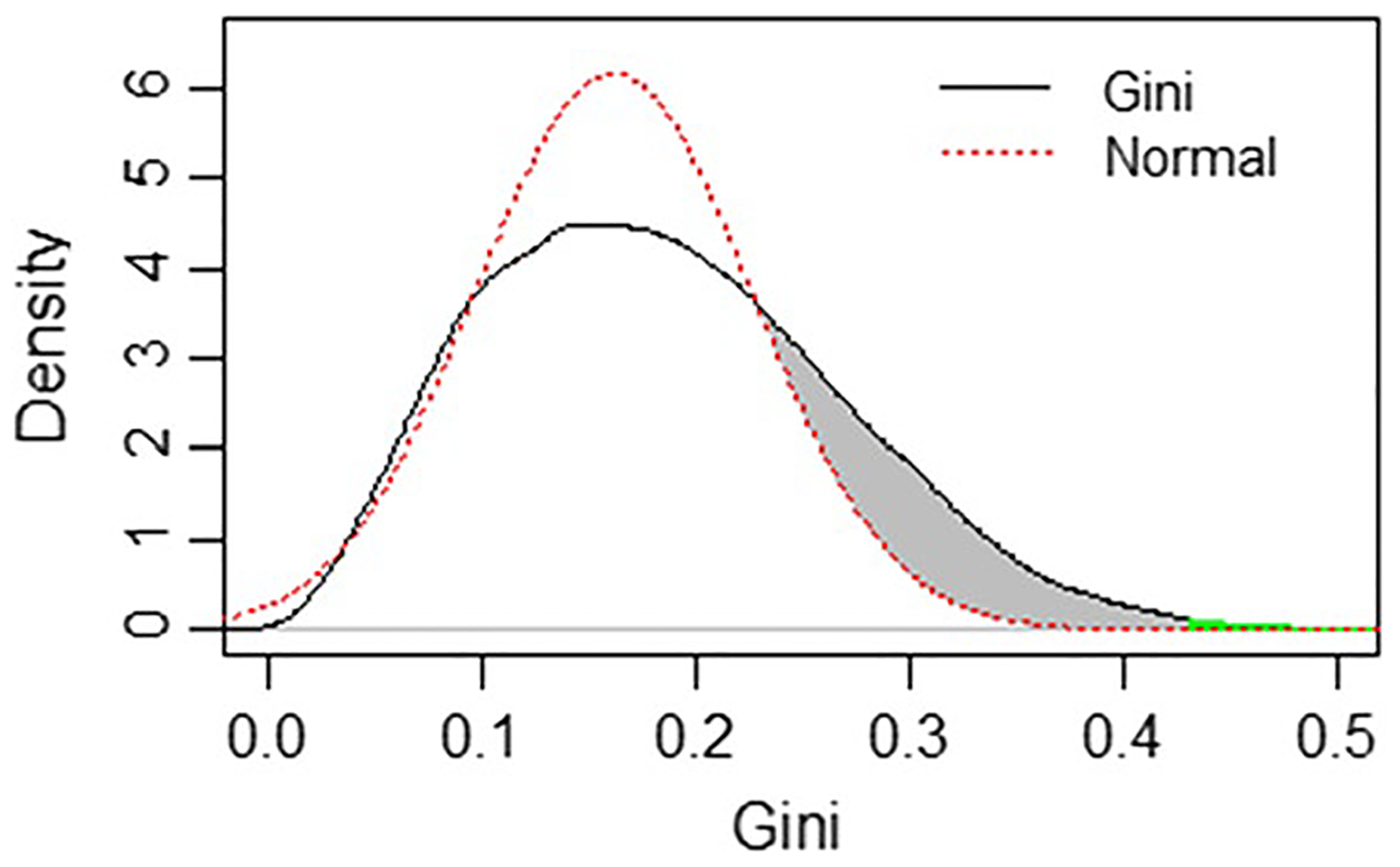
Gini index distribution (black curve) against its expected Gaussian counterpart (red curve). The gray area reflects the estimated excess of large Gini values. The green area marks the 81 extreme Gini values (FDR < 0.05)

The black line in Fig. 10 shows the distribution of Gini values for all authors, whereas the red dotted line shows a Gaussian distribution, with a mean and deviance that best fit the left-hand side of the curve. We then compute for each Gini number the p-value by which we can reject the null hypothesis that it is part of the reference distribution, correcting for false discovery rate (FDR) (Hochberg & Benjamini, 1990). Authors with the lowest FDR values will correspond to those who have an abnormally large NSC Gini index and by which the null hypothesis that such patterns are normal can be confidently rejected. The full list of authors and their FDR p-values is available upon request to the corresponding author. There are 81 authors (0.4%) with FDR < 0.05 and 231 with FDR < 0.10 (1.1%). Thus, the estimated prevalence of RLM is between 0.4% and 1.1%. Importantly, this estimate is well within the range of a study of 69,000 peer-review reports conducted by Elsevier to estimate the prevalence of reviewer coercion, which estimated 0.79% of reviews returned contained evidence of “clear misconduct” (Baas & Fennell, 2019).

Summing 1-FDR across the entire set, provides us with an estimate that 3,284 (16%) of the authors in our subset have higher Gini values than expected (Fig. 10, grey area). Note that this is a population-level statement, not a threshold to evaluate individual Gini scores. It suggests that about 16% of authors may have engaged to some degree, on one or more occasions, in RLM.

### Excessive self-citation suggests an author may be more likely to coerce others to cite their work

Because %SC ranking correlates so well with all the NSC-based metrics (Table 2), we examined whether or not %SC effectively represents a risk factor for RLM. A prior small-scale study of Norwegian authors found a correlation between SC and NSC (Fowler & Aksnes, 2007). They hypothesized the increase in NSC was due to an “advertising” effect caused by SC. However, an alternative hypothesis might be that authors that place a high value on citation of their work might be inclined to use all venues available to them, citing themselves and asking others to as well.

**Fig. 11:**
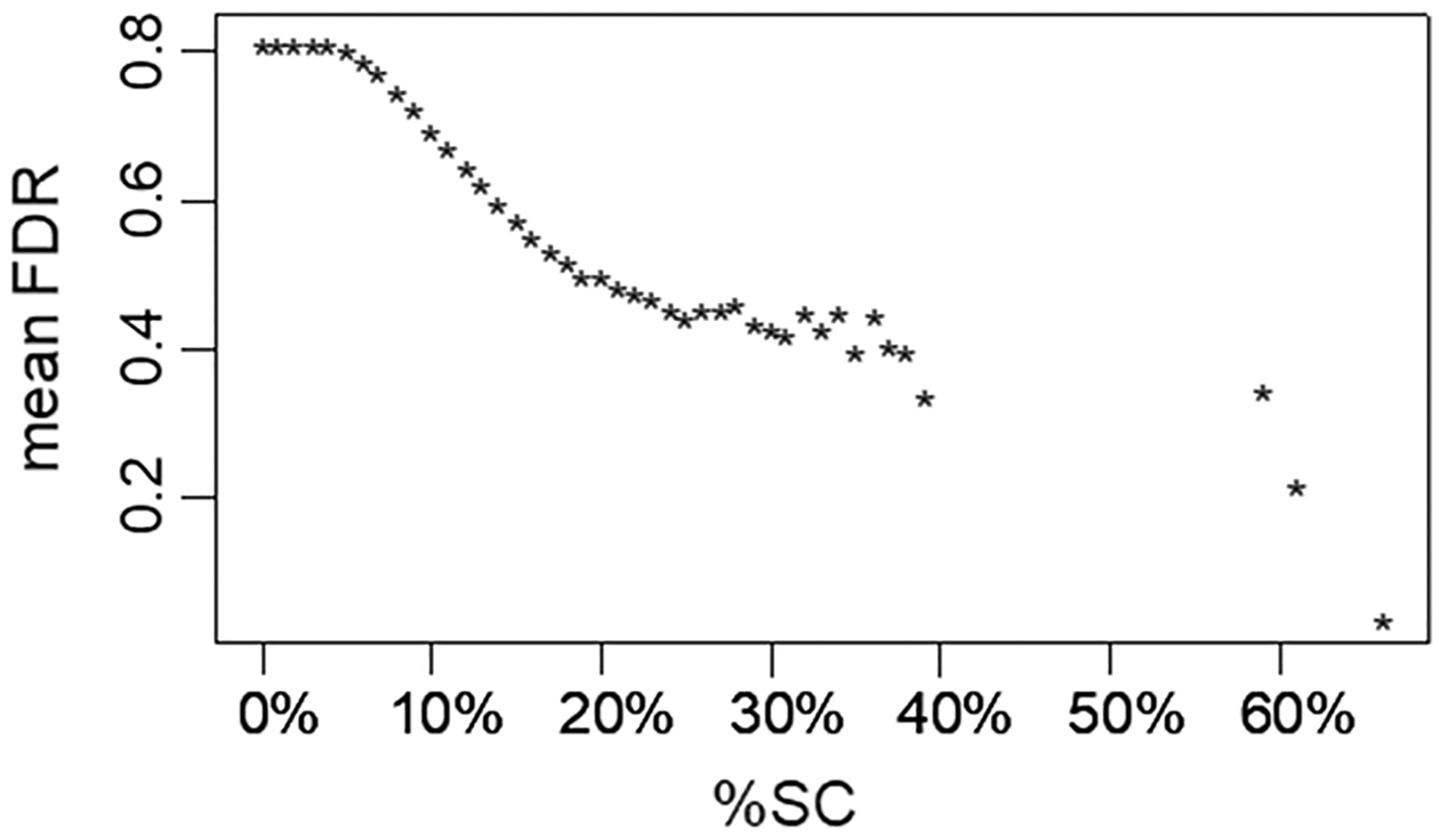
High %SC is correlated with signs of coercive behavior. For every author within each percentile of self-citation (x-axis), the mean Gini FDR (False Discovery Rate) is calculated for all authors above that percentile. Low Gini FDR values correspond to highly abnormal/skewed NSC patterns. If %SC were not predictive of NSC abnormalities, then the line would be approximately flat. Instead, the trend shows as %SC increases, authors greater than or equal to that amount have increasingly low Gini FDR values

Figure 11 shows that, as an author’s %SC rises, their NSC-based Gini index FDR value drops. This means the more of their reference list an author reserves for self-citation, the more distorted their single-paper NSC frequency distribution is. Interestingly, the figure shows the average FDR curve flattening around 20% SC, suggesting that the ability of %SC to predict coercive NSC behavior has reached a point of diminishing returns. Recursive partitioning identified ≥ 18% SC as the optimal threshold for the group, separating a set of 948 authors at almost 50% risk (average FDR < = 0.5), significantly higher than the rate for the group as a whole (t-test statistic = 37, p < 1e-16).

### Case studies

We provide a list of all authors analyzed, their Gini values and their red flag metrics in a supplementary Excel file which is available upon request to the corresponding author. It is important to note that numbers are calculated using the PMC citation network subset and will be different from the same calculations (e.g., total papers & citations) derived using different sources such as ISI or Google Scholar. Our goal in this section is not to judge guilt or innocence, but to illustrate how high Gini scores tend to be associated with multiple unusual patterns (“red flags”) suggestive of citation hacking, and to show how the red flag metrics lend themselves towards reasonable hypotheses regarding the potential origin of the distortion. We present one case suggestive of reviewer-coerced citation, one of editorial coercion or intervention, and one of author-author co-citation patterns that suggest mutual benefit.

The author with the highest Gini (Gini Rank #1), received 17 + NSC to his own papers 73 times, despite being in the 40th percentile in total NSC, and has the highest observed to expected 17 + NSC ratio (114) by far among all authors. His MCJ index is also the highest among all authors (2.87), which is due to 72 of these 73 papers all coming from one journal (*Surgical Endoscopy*). Examining a random subset of these papers, we find they are predominantly from commentaries on other papers published in the journal rather than research papers. He has the highest rank in multiple red flag categories. He has the highest ratio of consecutive blocks per NSC (0.664), and the presence of very large blocks (> 20) of consecutive citations were confirmed by manual examination of a random subset. He fell below threshold for fractional self-citation calculations with only 8 anchor author papers in the citation network, but averaged 32% self-citations in these 8. Interestingly, unusual self-citation and co-citation patterns for this author were reported in a prior study (Ioannidis, 2015), which hypothesized that such a pattern suggested he was attempting to raise his H-index. Combined, this is highly suggestive of editorial citation hacking.

The author with Gini Rank #2, ranks in the 12th percentile for total NSC, but received 17 + NSC to his work from 60 separate papers (Obs/Exp = 42), the 3^rd^ highest. An estimated 35% of the extracted references in the papers he authored are self-citations (rank = 19th out of 20,803). His MCJI suggests these distortions, in general, are not attributable to influence at one specific journal. He has the 7th highest number of blocks/NSC. Examining the context of the block citations in the published papers, we found a high degree of textual similarity surrounding them and that the context of the citations appears trivial (e.g., mentioning that user-friendly webservers are important followed by a very large number of citations to his papers). We also noticed that his name is mentioned frequently in the title of papers with excessive NSC, and that these have become increasingly frequent in recent years. Querying MEDLINE directly, we estimated almost 200 publications mention him by name in the title, but found only ~ 10% within our extracted citation network, suggesting the magnitude of his Gini distortion may be a significant underestimate. Googling textual phrases preceding large citation blocks to his papers (e.g., “to develop a really useful predictor”) shows that the same phrases appear in hundreds of papers verbatim as well as in a post-publication peer-review report online (MDPI_report, 2020). In this report, the reviewer requests 147 citations, the vast majority to this author, and after the first round of revision, rejects the paper because the authors did not accept the 1 ^st^-round request to change their title to include his name. This pattern is highly suggestive of reviewer-coerced citation, at least as a primary mechanism. However, we did observe an unusually large, but transient, surge of extreme NSC per paper coupled with his name being mentioned in the title of papers within two journals (*Prot Pept Let* from 2012–3 and *J Theor Biol* from 2018–9). We contacted *J Theor Biol* in 2019 because his activity was ongoing at the time, and an investigation by the editors in chief revealed that he had committed a number of ethical breaches as editor, including coercive requests to cite a large number of his papers (Chaplain et al., 2020).

The authors with Gini rank #3 and #8 cite each other extensively in papers they do not co-author, with #8 being the Most Citing Author (MCA) of #3 but not vice-versa. Having the same last name suggests they are related, the bylines in their papers shows they work at the same institution, the titles of their non-joint papers suggest they research the same subject, and PubMed shows they co-author frequently together. One of the reasons they may score so prominently is they also have a high rate of self-citation. A total of 19% and 20%, respectively, of their reference lists were self-citations, which puts them each in the top 3%. So on one hand, their NSC distortion could be attributed to a proclivity for self-citation plus entangled research activity but, on the other, it can be argued they each benefit from these co-citation patterns.

## Discussion

We present a novel method to detect people who may be regularly using their trusted position within the peer-review process, as well as author-author citation rings, to obtain significant additional citations to their work. Citation hacking takes place on the level of an individual paper submitted for publication, and our method uses the cumulative frequency of non-self-citations (NSC) a researcher receives from single papers to their body of published work. Because the frequency of NSC follows Zipf’s law, the statistical significance of deviations from this distribution can be estimated. The deviation is intended as a prioritization method, designed not to catch individual instances but examine trends.

Some of the limitations of this study should be noted. First, the citation network we have is limited to PubMed and only a subset of the whole. References to papers will be biased towards the types of full-text articles that are most likely to be deposited in PubMed Central. Since the United States government mandates that publications resulting from federally funded research be deposited there, it will likely be biased towards authors in the US. Studies originating in and funded by other countries will likely be under-represented in the PMC citation network. Second, although our heuristics for author name disambiguation work reasonably well in general, they are imperfect and it biases our author subset towards authors with less ambiguous names. Consequently, authors with a national origin in which names are more ambiguous would be under-represented in this subset, so drawing demo-graphic conclusions would be problematic. Third, the order in which citations appear in the XML files versus the PDFs is not preserved with high fidelity, so our calculations will underestimate the actual frequency and size of contiguous citation blocks to each author. Fourth, it is possible that the incentive to engage in RLM is higher for newer authors than well-published authors simply because the proportional gain per attempt will be higher. Finally, the study focuses on currently active authors and may not be representative of highly productive authors throughout PubMed’s entire history.

Prior to this study, citation hacking had only been studied via surveys and anecdotal reports. Prior algorithmic approaches focused specifically on journal-based citation hacking. The number of currently active authors that we can declare, with 95% confidence, have significantly distorted NSC distributions was between 0.4% and 1.1%, an estimate that concurs well with a manual evaluation of 69,000 peer-review reports (0.79%) (Baas & Fennell, 2019). Although this suggests the fraction of authors that may have engaged in chronic citation hacking is relatively small, they have also been highly active, meaning the percent of authors who have been hacked is much higher. Furthermore, the difference between the actual and ideal Gini curves suggests approximately 16% of authors have engaged in RLM to some degree, possibly opportunistically and not necessarily egregiously.

Our analysis highlights the importance of having some system in place to detect and prevent citation hacking efforts before they become part of the published record. Once they do, it is unclear what should be done with compromised papers. One solution, would be to remove coerced references once detected, even post-publication. Unfortunately, that may not be logistically simple for publishers and indexers and authors may be reluctant to cooperate if they feel admitting coercion might either tarnish their paper or reputation. Another would be to create a database of reviewer/editor-added citations, which could then be subtracted out of calculated metrics such as the H-index, but this would be cumbersome and incomplete. Perhaps the simplest solution would be to cap the number of citations (SC or NSC) per paper used for author-based or organization-based metric calculations, such as the H-index. This would eliminate even the need to detect anomalies, but would require gathering extra information not provided explicitly within reference lists (e.g., finding all author names omitted by ‘*et. al*.’).

## Conclusion

If citations are the currency of science, then citation hacking is akin to counterfeiting. Trust in a currency is integral to its value and continued use. If we, as a scientific community, plan to continue using citations as a metric that reflects influence, then we need to be able to detect when the system may have been gamed. There are advantages to having a decentralized scientific publishing system, but one disadvantage is an inability to share information about bad actors within the system. Similarly, anonymous peer-review enables honest assessment without fear of repercussions, but also cloaks self-interested behavior. We cannot rely upon anecdotal reports coming from journals to identify bad actors, particularly if they are reluctant to name them (Chaplain et al., 2020; Wren et al., 2019), because our data has shown cases of potential concern that started long ago and are still ongoing as of this writing. We’ve attempted to find the best solution within our grasp: A straightfor-ward method to detect and prioritize; an unbiased study of all highly active, well-published authors; and the ability to put single instances of potential misconduct within a broader, historical context for each author.

Along these lines we caution that, although it is almost inevitable that the efforts of citation hackers will leave “fingerprints” within the citation record, it does not necessarily follow that all authors with a high NSC Gini Index obtained them through unethical or coercive means. The NSC Gini Index merely permits the problem to be framed as a null hypothesis test, whereby the statistical significance of an author’s deviation from the norm can be quantitatively established. It does not specify *why* it deviates. A manual investigation of papers with anomalously high NSC should be conducted prior to accusing an author of RLM.

## Supplementary Material

Supplementary Table S1

Supplementary Figures

## Figures and Tables

**Table 1:** Summary of red flags used to identify patterns of behavior that are suggestive of RLM

Red Flag #	Abbr	For each author, an unusually high frequency of
1	Blocks	Consecutive NSC to an author within papers
2	NSCI	H-index for papers with multiple NSC to an author
3	17 +	# of papers with extreme NSC events
4	MCJI	Journal-specific NSC H-index for an author
5	%SC	% of reference list used for self-citation

**Table 2:** Spearman’s rank correlation among “red flags” that are suggestive of citation hacking

	Gini	NSCI	Blocks	17 +	MCJI	%SC	Factor
Gini	1	0.82	0.63	0.48	0.68	0.55	**0.95**
NSCI	0.82	1	0.66	0.52	0.61	0.52	0.93
Blocks	0.63	0.66	1	0.43	0.47	0.51	0.75
17 +	0.48	0.52	0.43	1	0.39	0.35	0.58
MCJ	0.68	0.61	0.47	0.39	1	0.38	0.73
%SC	0.55	0.52	0.51	0.35	0.38	1	0.63

Blocks = citations in contiguous blocks (> = 3); 17 + = Papers with 17 or more NSC to one author

*MCJI* Most Citing Journal Index; *Factor* aggregate factor analysis score

*Gini* Gini index of NSC distribution; *NSCI* non-Self Citation Index; %*SC* average percent of the reference list used for self-citation

Notably, Gini has the highest predictive power (bold), explaining
