# Supplementary material for: Detecting anomalous referencing patterns in PubMed papers suggestive of author-centric reference list manipulation
Source: Scientometrics. Author manuscript; Available in PMC 2024 Feb 2. (PMC10836843; doi:10.1007/s11192-022-04503-6)
Supplement: Supplementary Figures [file NIHMS1921645-supplement-Supplementary_Figures.pdf]

Supplementary Figure 1

Figure Title: Correlations between factor analysis and the other 6 Red Flag metrics

Figure Legend: A comparison of the correlations of factor analysis as a means of flagging suspicious patterns within reference lists with the other 6 Red Flag metrics. Each of the density plots shows the distribution of values for one Red Flag (y-axis) versus the others (x-axis).

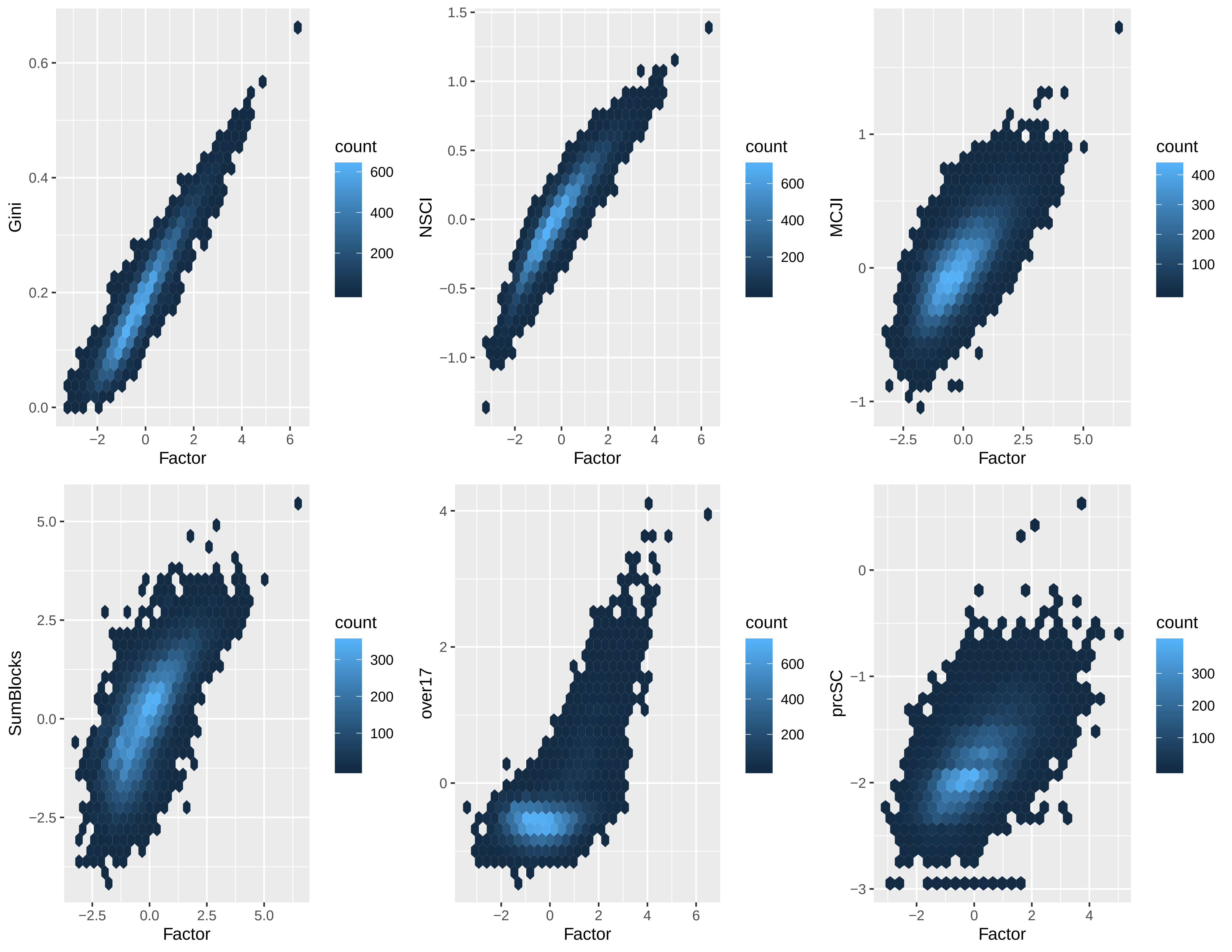
Gini
Factor
count
600
400
200
NSCI
Factor
count
600
400
200
MCJI
Factor
count
400
300
200
100
SumBlocks
Factor
count
300
200
100
over17
Factor
count
600
400
200
prcSC
Factor
count
300
200
100

Supplementary Figure 2

Figure Title: Correlations between Gini score and the other 6 Red Flag metrics

Figure Legend: A comparison of the correlations of the Gini score as a means of flagging suspicious patterns within reference lists with the other 6 Red Flag metrics. Each of the density plots shows the distribution of values for one Red Flag (y-axis) versus the others (x-axis).

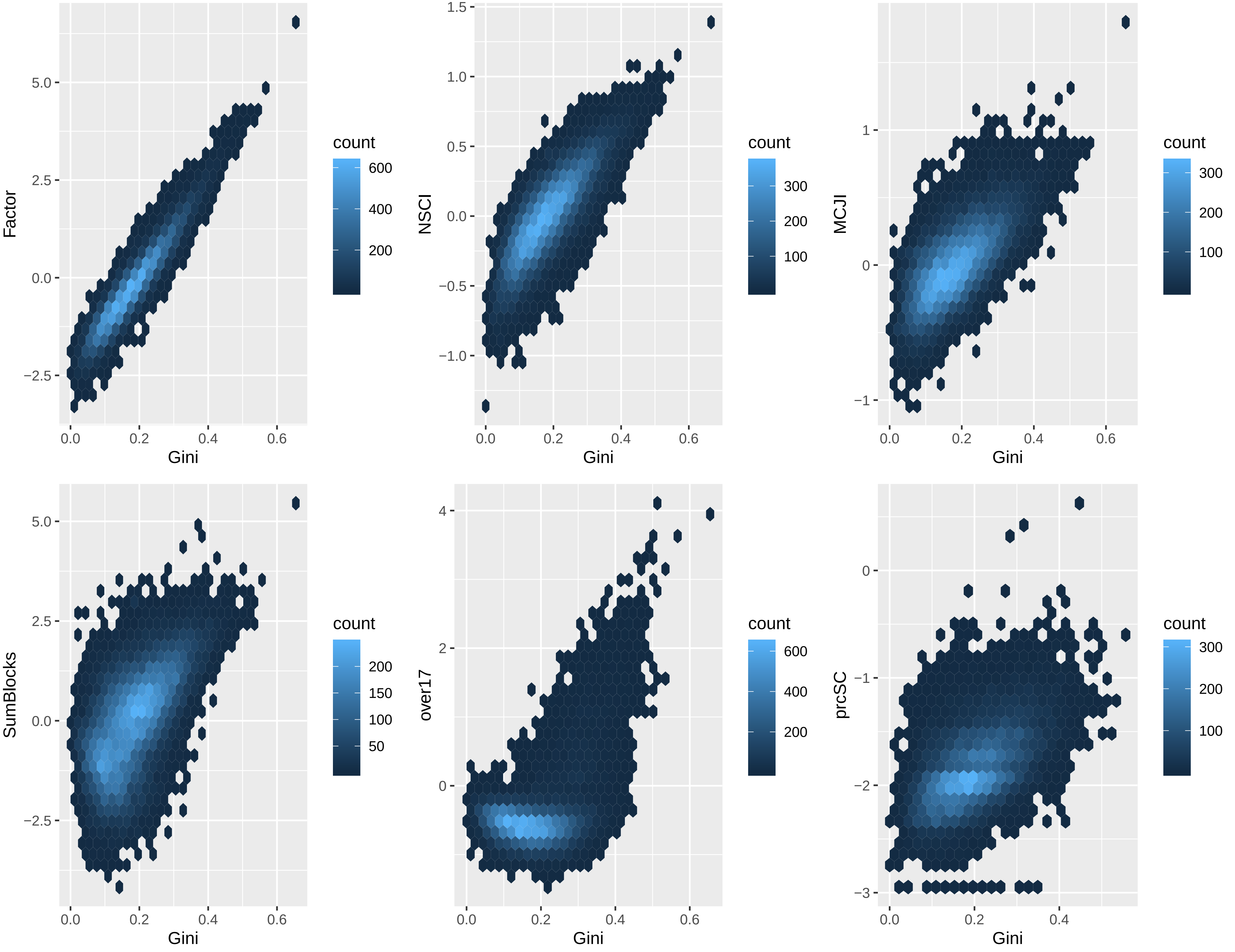

Factor
count
600
400
200
5.0
2.5
0.0
−2.5
0.0
0.2
0.4
0.6
Gini
NSCI
count
300
200
100
1.5
1.0
0.5
0.0
−0.5
−1.0
Gini
MCJI
count
300
200
100
1
0
−1
Gini
SumBlocks
count
200
150
100
50
5.0
2.5
0.0
−2.5
Gini
over17
count
600
400
200
4
2
0
Gini
prcSC
count
300
200
100
0
−1
−2
−3
Gini

Supplementary Figure 3

Figure Title: Correlations between the Most Cited Journal Index (MCJI) and the other 6 Red Flag metrics

Figure Legend: A comparison of the correlations of the MCJI as a means of flagging suspicious patterns within reference lists with the other 6 Red Flag metrics. Each of the density plots shows the distribution of values for one Red Flag (y-axis) versus the others (x-axis).

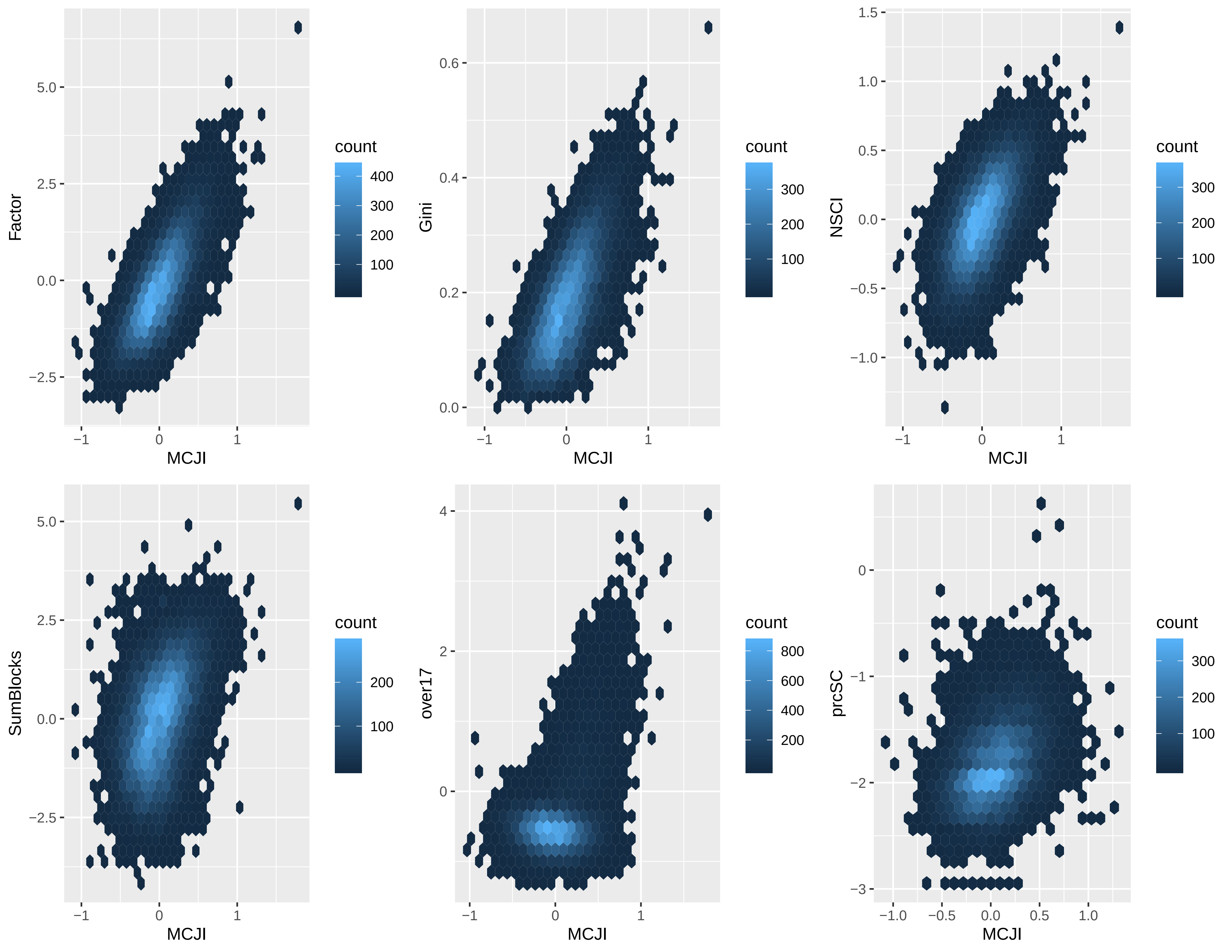

Factor
MCJI
count
400
300
200
100
Gini
MCJI
count
300
200
100
NSCI
MCJI
count
300
200
100
SumBlocks
MCJI
count
200
100
over17
MCJI
count
800
600
400
200
prcSC
MCJI
count
300
200
100

Supplementary Figure 4

Figure Title: Correlations between the Non-Self Citation Index (NSCI) and the other 6 Red Flag metrics

Figure Legend: A comparison of the correlations of the NSCI as a means of flagging suspicious patterns within reference lists with the other 6 Red Flag metrics. Each of the density plots shows the distribution of values for one Red Flag (y-axis) versus the others (x-axis).

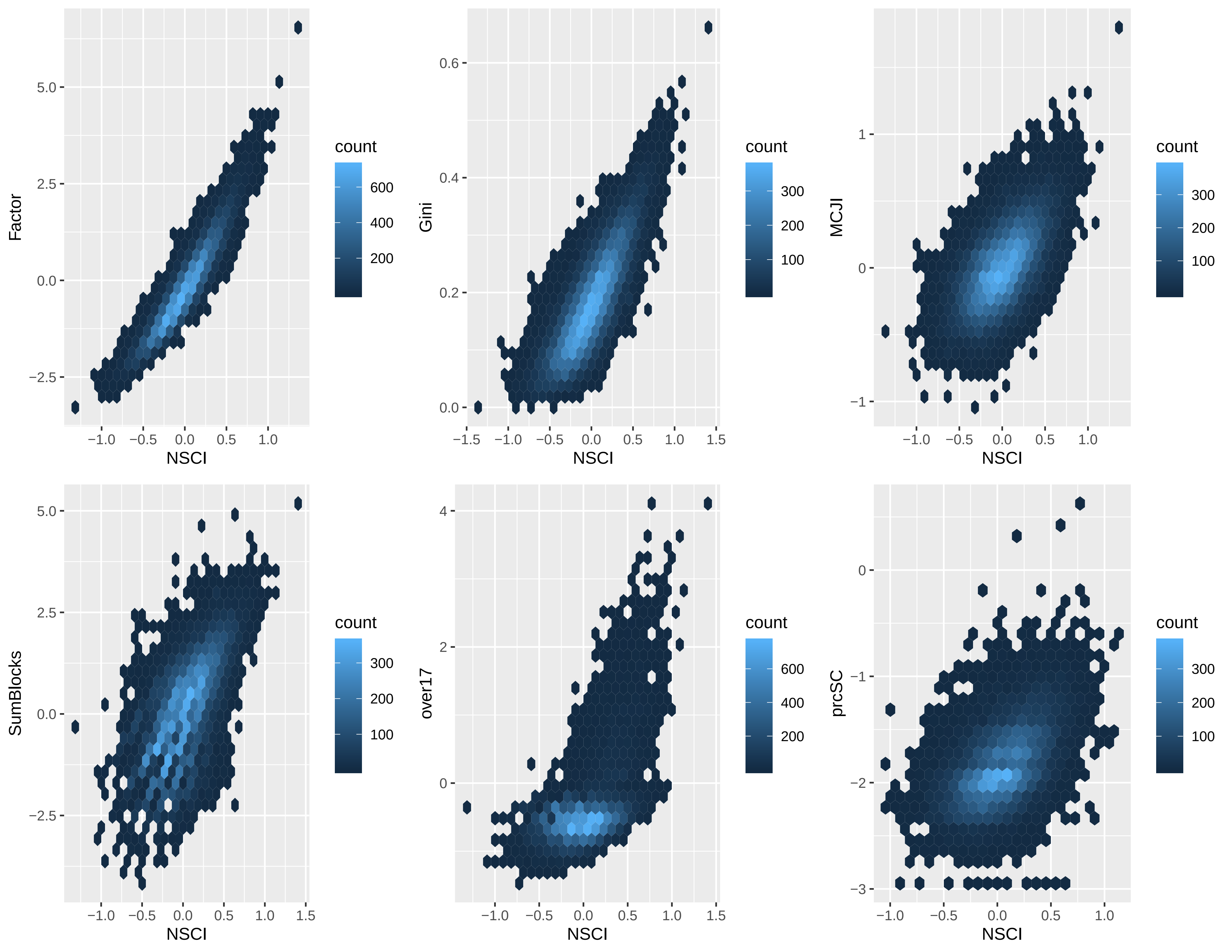
Factor
NSCI
count
Gini
MCJI
SumBlocks
over17
prcSC

Supplementary Figure 5

Figure Title: Correlations between the number of 17+ Non-Self Citations Observed to the number Expected (17+ Obs/Exp) and the other 6 Red Flag metrics

Figure Legend: A comparison of the correlations of the 17+ Obs/Exp as a means of flagging suspicious patterns within reference lists with the other 6 Red Flag metrics. Each of the density plots shows the distribution of values for one Red Flag (y-axis) versus the others (x-axis).

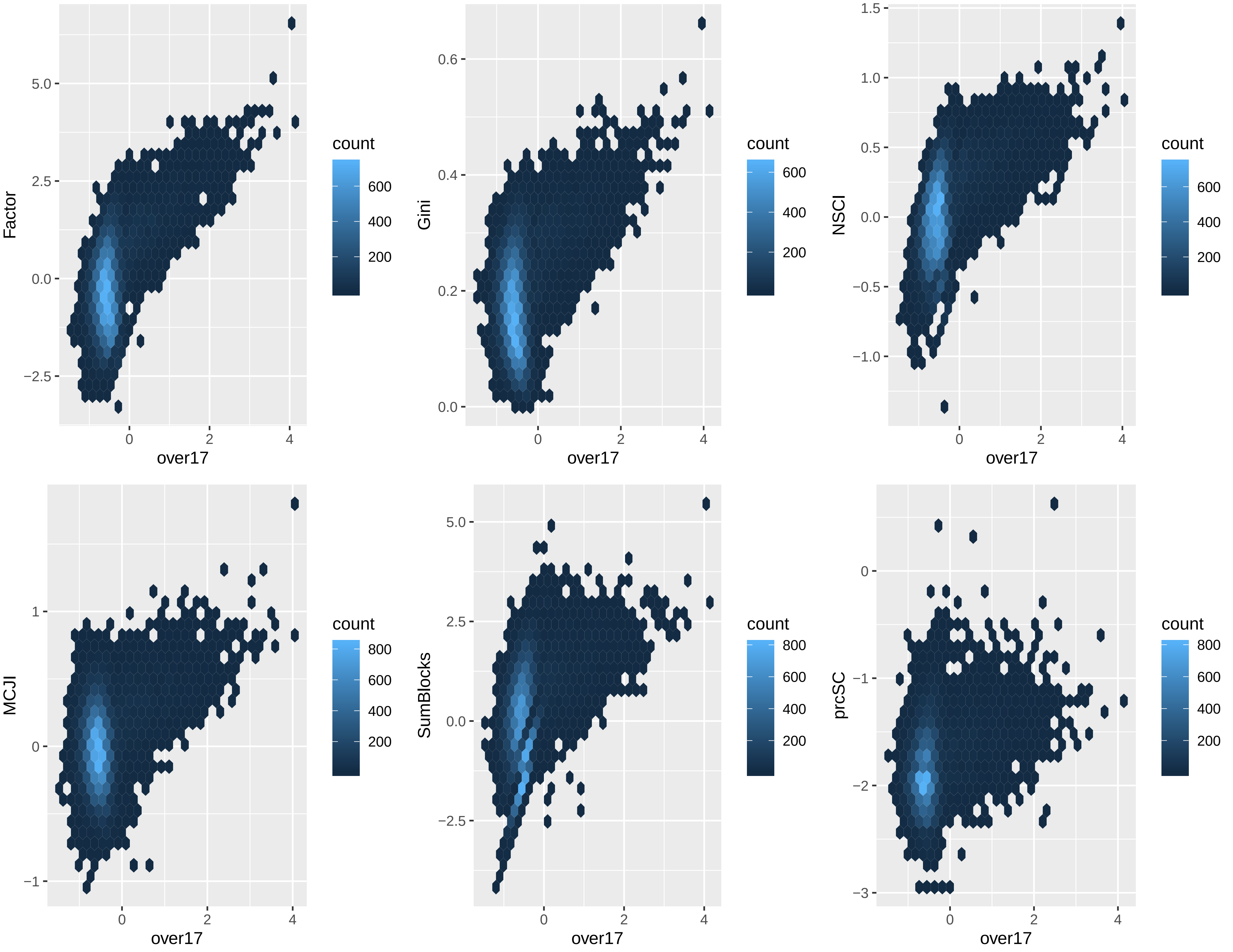
Factor
5.0
2.5
0.0
−2.5
0
2
4
over17
count
600
400
200
Gini
0.6
0.4
0.2
0.0
0
2
4
over17
count
600
400
200
NSCI
1.5
1.0
0.5
0.0
−0.5
−1.0
0
2
4
over17
count
600
400
200
MCJI
1
0
−1
0
2
4
over17
count
800
600
400
200
SumBlocks
5.0
2.5
0.0
−2.5
0
2
4
over17
count
800
600
400
200
prcSC
0
−1
−2
−3
0
2
4
over17
count
800
600
400
200

Supplementary Figure 6

Figure Title: Correlations between the percent of the reference list used for Self-Citation (%SC) and the other 6 Red Flag metrics

Figure Legend: A comparison of the correlations of the %SC as a means of flagging suspicious patterns within reference lists with the other 6 Red Flag metrics. Each of the density plots shows the distribution of values for one Red Flag (y-axis) versus the others (x-axis).

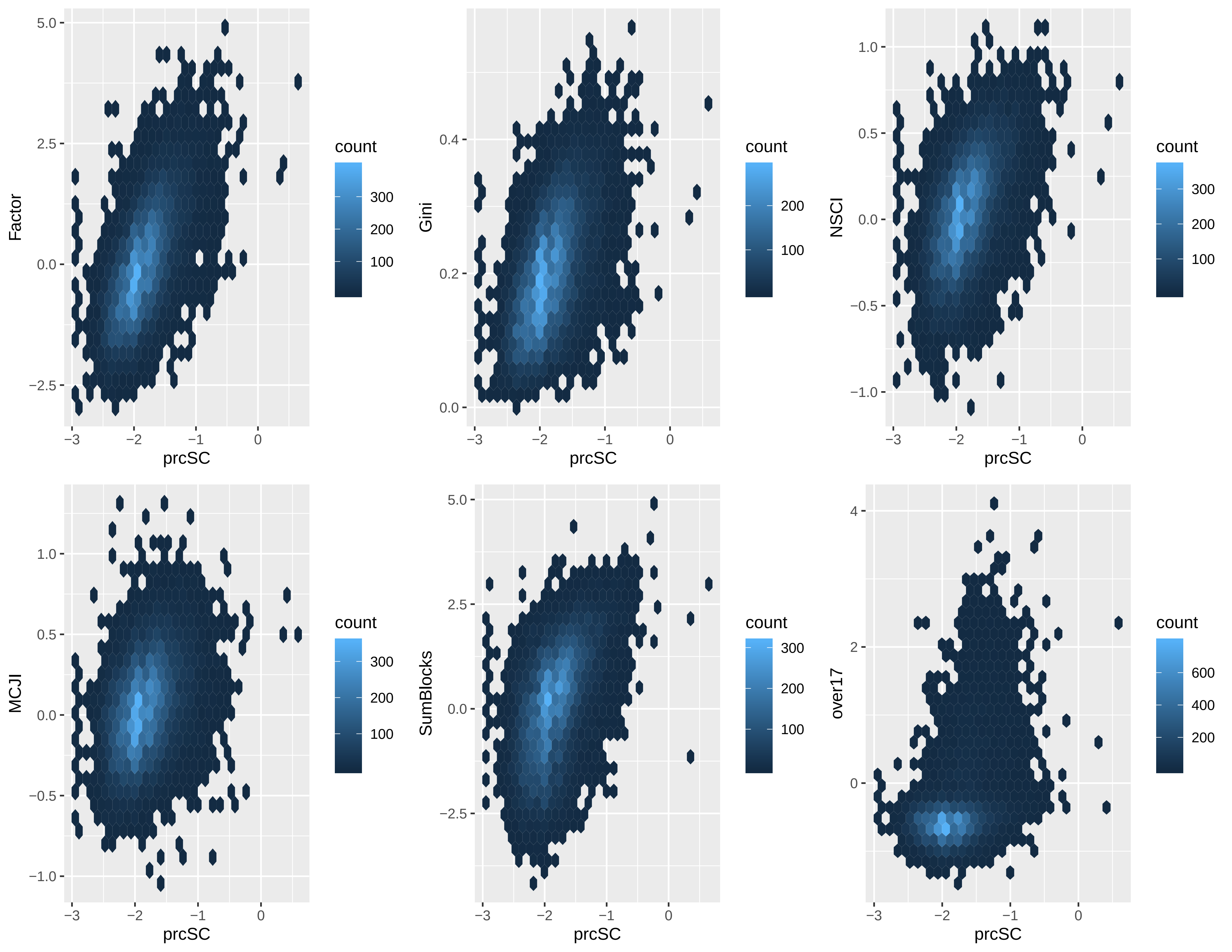

Factor
prcSC
count
300
200
100
Gini
prcSC
count
200
100
NSCI
prcSC
count
300
200
100
MCJI
prcSC
count
300
200
100
SumBlocks
prcSC
count
300
200
100
over17
prcSC
count
600
400
200

Supplementary Figure 7

Figure Title: Correlations between the number of blocks of consecutive Non-Self Citations found within the reference list (NSCblocks) and the other 6 Red Flag metrics

Figure Legend: A comparison of the correlations of the NSCblocks as a means of flagging suspicious patterns within reference lists with the other 6 Red Flag metrics. Each of the density plots shows the distribution of values for one Red Flag (y-axis) versus the others (x-axis).

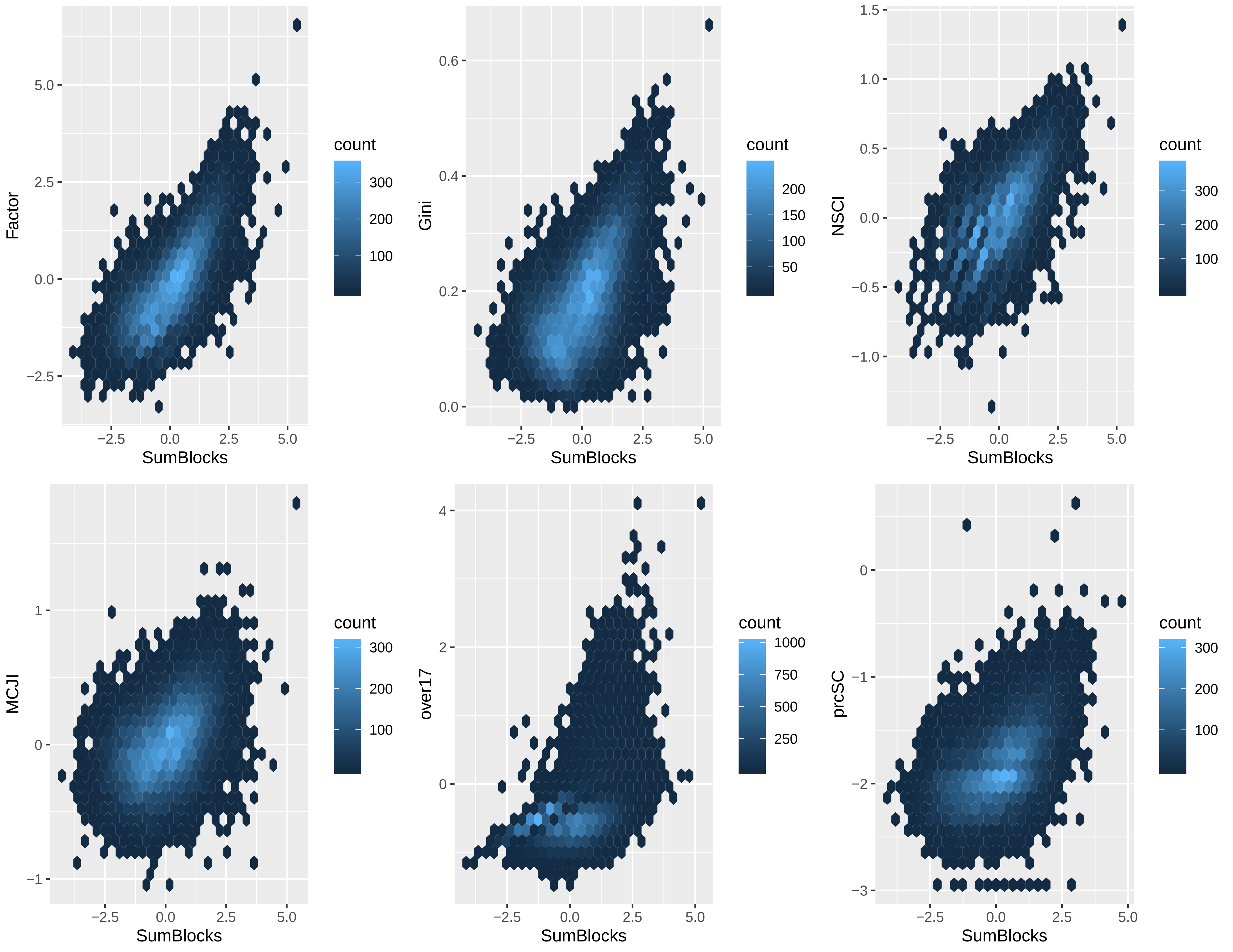
Factor
SumBlocks
count
Gini
SumBlocks
count
NSCI
SumBlocks
count
MCJI
SumBlocks
count
over17
SumBlocks
count
prcSC
SumBlocks
count
